# Coordinated Interactions between the Hippocampus and Retrosplenial Cortex in Spatial Memory

**DOI:** 10.34133/research.0521

**Published:** 2024-10-31

**Authors:** Ruiqing Hou, Ziyue Liu, Zichen Jin, Dongxue Huang, Yue Hu, Wenjie Du, Danyi Zhu, Leiting Yang, Yuanfeng Weng, Tifei Yuan, Bin Lu, Yingwei Wang, Yong Ping, Xiao Xiao

**Affiliations:** ^1^ Department of Anesthesiology, Huashan Hospital; Key Laboratory of Computational Neuroscience and Brain-Inspired Intelligence, Ministry of Education; Behavioral and Cognitive Neuroscience Center, Institute of Science and Technology for Brain-Inspired Intelligence, MOE Frontiers Center for Brain Science, Fudan University, Shanghai 200433, China.; ^2^ School of Life Science, Fudan University, Shanghai 200032, China.; ^3^ Shanghai Key Laboratory of Psychotic Disorders, Brain Health Institute, National Center for Mental Disorders, Shanghai Mental Health Center, Shanghai Jiaotong University School of Medicine, Shanghai 200030, China.; ^4^ Department of Endocrinology and Metabolism, Huadong Hospital, Fudan University, Shanghai 200040, China.; ^5^ Bio-X Institutes, Key Laboratory for the Genetics of Developmental and Neuropsychiatric Disorders (Ministry of Education), Shanghai JiaoTong University, Shanghai 200240, China.

## Abstract

While a hippocampal–cortical dialogue is generally thought to mediate memory consolidation, which is crucial for engram function, how it works remains largely unknown. Here, we examined the interplay of neural signals from the retrosplenial cortex (RSC), a neocortical region, and from the hippocampus in memory consolidation by simultaneously recording sharp-wave ripples (SWRs) of dorsal hippocampal CA1 and neural signals of RSC in free-moving mice during the delayed spatial alternation task (DSAT) and subsequent sleep. Hippocampal–RSC coordination during SWRs was identified in nonrapid eye movement (NREM) sleep, reflecting neural reactivation of decision-making in the task, as shown by a peak reactivation strength within SWRs. Using modified generalized linear models (GLMs), we traced information flow through the RSC–CA1–RSC circuit around SWRs during sleep following DSAT. Our findings show that after spatial training, RSC excitatory neurons typically increase CA1 activity prior to hippocampal SWRs, potentially initiating hippocampal memory replay, while inhibitory neurons are activated by hippocampal outputs in post-SWRs. We further identified certain excitatory neurons in the RSC that encoded spatial information related to the DSAT. These neurons, classified as splitters and location-related cells, showed varied responses to hippocampal SWRs. Overall, our study highlights the complex dynamics between the RSC and hippocampal CA1 region during SWRs in NREM sleep, underscoring their critical interplay in spatial memory consolidation.

## Introduction

Memory consolidation, crucial in engram formation, involves storing and integrating new information into existing long-term memories, thereby supporting the information recall [1,2]. It is widely believed that hippocampal–cortical dialogue underlies this process [3–6]. However, there are no clear differences in the content of hippocampal–cortical information interaction between awake and sleep states [1,7]. Some theories of memory consolidation focus on the interaction between the neocortex and the hippocampus during sleep, where repeated reactivation of hippocampal representations drives neocortical plasticity, promoting the stabilization of long-term memory [8–10]. Experiences during wakefulness not only influence hippocampal neural activities [11,12] but also shape hippocampal–cortical [13] or hippocampal–subcortical [14] dialogues during sleep, supporting the hypothesis.

The RSC, situated in the midline of the brain, reveals reciprocal connections with hippocampal formation [15,16]. Both the hippocampus and RSC are indispensable for spatial cognition and memory [12,17–25]. Lesions in hippocampus impair the development of the RSC to encode spatial information[26], while temporary inhibition of RSC activity affects the stability of hippocampal place cells [27]. These studies suggest that the hippocampal–RSC interaction is essential for generating stable representations. During active wakefulness and rapid eye movement (REM) sleep, hippocampal theta rhythms are coupled with RSC gamma activity [28,29]. Moreover, a fluctuated interaction strength in the hippocampus and RSC during phasic REM played a functional role in hippocampal–cortical memory consolidation [30]. In contrast, studies on nonrapid eye movement (NREM) sleep have uncovered fewer insights into hippocampal–RSC interactions. Recent evidence suggests a direct dorsal hippocampal–RSC connection through optogenetic stimulation, which activates RSC inhibitory neurons [31]. However, the precise mechanisms by which hippocampus–RSC interaction occurs during NREM sleep, specifically concerning information transfer and integration, remain poorly understood.

During NREM sleep, a hippocampal oscillation, known as sharp-wave ripples (SWRs), is regarded as a cognitive biomarker for memory processing [4,12,17]. Coordination of hippocampal SWRs and cortical patterns during NREM sleep has been hypothesized as a potential mechanism of hippocampal–cortical communication to sustain memory consolidation [1,3,5,6]. Enhancing hippocampal–cortical coordination through generating artificial cortical delta waves or spindles after SWRs improved performance on memory tasks [13,32], suggesting the importance of the cooperation between hippocampal SWRs and cortical low-frequency oscillations for memory consolidation. Further, the ensemble spiking of the hippocampus during SWRs can predict subsequent cortical firing patterns [10,33]. Recent studies have uncovered high-frequency ripple oscillations in the rat association cortex, notably suggesting the coupling of RSC ripples with hippocampal ripples [34,35]. These results show that there may be cortical–hippocampal–cortical circuits during SWRs that facilitate information transduction for memory during SWRs in NREM sleep. However, the directionality of information flow between the hippocampus and RSC during the SWRs, a key marker of memory processing, remains unclear.

In this study, we recorded electrophysiological signals in dorsal hippocampal CA1 and RSC during 3 different phases: the training of a spatial alternation task, the pre-training sleep phase (pre-sleep), and the post-training sleep phase (post-sleep). Our goal is to elucidate the neural dialogue between the RSC and hippocampus during sleep, specifically focusing on the period around SWRs (pre-SWRs, within SWRs, post-SWRs), and to determine how this interaction influences spatial memory processing.

## Results

### Bilateral RSC lesions induced deficits in memory retrieval but not in task learning

**Fig. 1.**
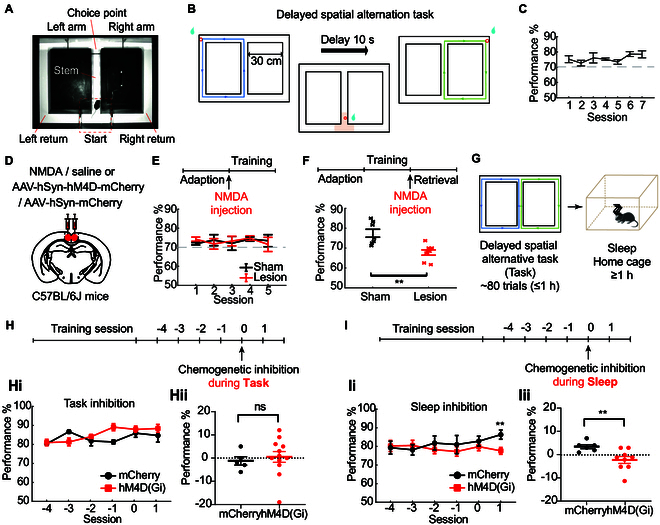
Effects of RSC manipulation on DSAT. (A) Experimental apparatus of the figure-eight maze used in the experiments. (B) Task paradigm. Mice were trained in a DSAT where they navigated the central stem each trial, making alternate left and right choices at the T junction. Correct choices were rewarded at water ports at the end of each arm (red circles). Following each trial, mice were delayed for 10 s in the “start” area, where they received a reward for correct trials, before starting the next trial. (C) Performance of mice (*n* = 5), with beginner defined as accuracy exceeding 70% and expert defined as accuracy >75% in at least 7 consecutive days. Data are presented as mean ± SEM. (D) Schematic of NMDA or chemogenetic virus injection in RSC. (E) Top: Timeline for NMDA-induced RSC lesion induction prior to learning. Bottom: Training phase performance post-lesion (lesion: *n* = 5, sham: *n* = 5; *P* > 0.05, 2-way ANOVA). (F) Top: Timeline for NMDA-induced RSC lesion post-learning. Bottom: Plot of retrieval performance following lesion. Individual performance for control and lesioned mice (lesion: *n* = 8, sham: *n* = 7) is depicted with forks. **P < 0.01, Wilcoxon rank-sum test. (G) Schedule for the retrieval session. Neurophysiological data were recorded during both the spatial task and sleep phases. (H) Chemogenetic inhibition of RSC was applied during the task on day 0 (D0), following training until performance accuracy stabilized. (Hi) Performance across 6 sessions (D−4 to D1) for control (*n* = 5) and experimental (*n* = 12) groups. (Hii) Performance change in the session post-CNO injection relative to the prior session (D1 to D0) for both groups (Wilcoxon rank-sum test, *P* = 0.3681). (I) Chemogenetic inhibition of RSC was applied during post-task sleep on day 0, after training until performance accuracy stabilized. (Ii) Performance across 6 sessions (D−4 to D1) for control (*n* = 6) and experimental (*n* = 8) groups. (Iii) Performance change in the session post-CNO injection relative to the prior session (D1 to D0) for both groups (Wilcoxon rank-sum test, *P* = 0.0058).

A modified T maze with fully automatic doors and return arms, as a figure-8 maze, was used for the delayed spatial alternation task (DSAT) in mice (Fig. 1A). In the DSAT, water-restricted mice were trained trial by trial to alternate between the left and right sides to receive diluted sucrose water as a reward. When mice returned to the “start” area, they waited for a 10-s delay until the center door opened, which started the next trial, approaching to the side arm across the stem (Fig. 1A and B). Mice received 60 trials per day in DSAT training. Beginner was defined as accuracy exceeding 70%. Expert performance was defined as reaching an accuracy of above 75% in 7 consecutive sessions or performing correctly at 8 or more consecutive alternating trials in 2 sessions (Fig. 1C and Fig. S1B). To verify the function of the RSC, we performed neurotoxic lesions by stereotaxic injection of a high dose of *N*-methyl-d-aspartate (NMDA) into bilateral RSC (Fig. 1D and Fig. S1A). When lesions were performed before behavioral training (after 1-d habituation), it had no impact on the following performance in DSAT learning [Fig. 1E, 2-way analysis of variance (ANOVA), *F*(1,40) = 0.06, *P* = 0.81 for lesion, *F*(4,40) = 0.15, *P* = 0.96 for session]. However, when lesions were performed on the first day after the animals achieved expert level (Fig. S1C), it had a moderate but significant impairment on memory retrieval (Fig. 1F, *P* = 0.0019, Wilcoxon rank-sum test). These results indicate that the post-training lesions of the RSC may affect memory consolidation by impairing memory recall processes.

### Silencing RSC neurons during sleep impairs memory consolidation during retrieval

To further investigate the contribution of RSC in memory retrieval, we utilized the designer receptor exclusively activated by designer drugs (DREADD)-based chemogenetic tools and applied them during either the task or sleep phase of the retrieval session (Fig. 1G). The mice were bilaterally injected with inhibitory DREADD virus [AAV2/9-hSyn-hM4D(Gi)-mCherry] or control virus (AAV2/9-hSyn-mCherry) in the RSC (Fig. 1D and Fig. S1D). After the training session in the DSAT, when the virus-injected mice achieved expert performance level (>75% accuracy) in at least 7 consecutive days, they were administered clozapine *N*-oxide (CNO) by intraperitoneal injection during the task or sleep phase in the retrieval session 0 (Fig. 1H and I). The chemogenetic inhibition of RSC during task period did not significantly impact the performance accuracy in the DSAT task [Fig. 1Hi, 2-way ANOVA, *F*(1,88) = 1.943, *P* = 0.1668]. A comparison of performance between session 0 and the following day’s session 1 revealed that the hM4D(Gi) group’s DSAT execution was comparable to the control (mCherry) group (Fig. 1Hii, Wilcoxon rank-sum test, *P* = 0.3681). However, CNO administration during the post-DSAT sleep phase resulted in significant next-day performance impairments in the hM4D(Gi) group compared to the mCherry group [Fig. 1Ii and Iii, 2-way ANOVA, *F*(1,70) = 2.464, *P* = 0.1210, for Ii; Wilcoxon rank-sum test, *P* = 0.0058, for Iii], suggesting that inactivating RSC neurons during the sleep phase disrupts memory consolidation during the hypothetical memory rehearsal process that occurs during the ripples [36].

### Coordinated hippocampal SWRs and RSC oscillations during sleep

**Fig. 2.**
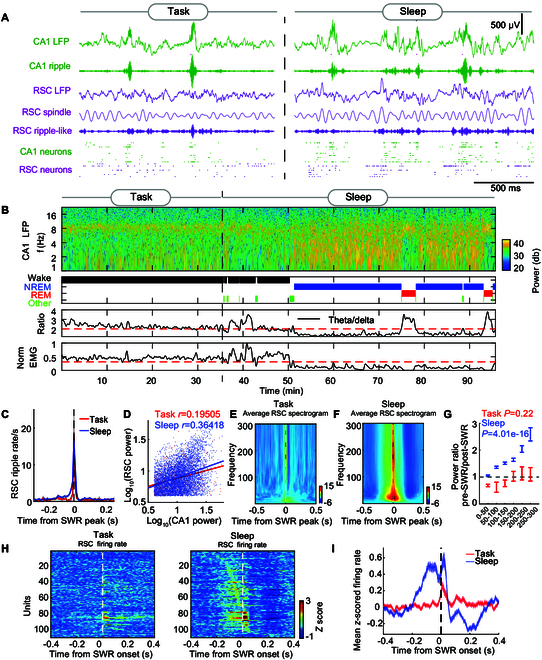
Association of RSC activity with hippocampal SWRs during sleep. (A) Representative simultaneously recorded CA1 and RSC LFPs (green and violet, respectively), band-pass filtered oscillations (ripple/ripple-like: 140 to 250 Hz, RSC spindle: 10 to 20 Hz), and CA1 and RSC spike trains during the spatial task (left) and sleep (right) phase, respectively. (B) Spectrogram and signals were used to define brain states (wake, NREM, or REM sleep). Top, spectrogram in CA1; middle top, hypnogram; middle bottom, the theta/delta ratio; bottom, the normalized EMG signal. (C) Average cross-correlogram between CA1 SWR peaks and RSC ripple-like event peaks during the task and the sleep phase. (D) Correlation of peak ripple band power, assessed by Pearson’s coefficient, between CA1 ripples and RSC ripple-like events during spatial task performance and the sleep phase. (E) Average RSC spectrogram aligned with CA1 SWR peaks during the spatial task, standardized using a *z*-score based on the mean and SD from −1 to −0.5 s prior to SWR. (F) Corresponding analyses to (E) for CA1 and RSC neural data during the sleep phase. (G) Frequency band analysis of pre-SWR (−100 ms to 0 ms) to post-SWR (0 to 100 ms) RSC spectrogram ratios during the spatial task and the sleep phase. A one-way ANOVA showed that RSC oscillation power across the slow-to-fast oscillation bands (50 to 300 Hz) shifted from pre-SWR (−100 ms to 0 ms) to post-SWR (0 to 100 ms) during sleep. (H). Heatmaps of *z*-scored SWR-correlated spiking activity of RSC neurons during the spatial task (left) and sleep (right), sorted by peak firing timing within a ±300-ms window around SWRs. All *z*-score transformations within the article utilize a baseline mean and SD from −1 to −0.5 s. (I) Mean *z*-scored PSTH of RSC neurons (*n* = 111 units) in relation to SWR onsets during the spatial task (red) and sleep (blue). Shown in mean ± SEM.

To further investigate the neural mechanism of memory consolidation processing in RSC, multi-channel electrophysiological recordings in dorsal hippocampal CA1 and RSC in mice were carried out (Fig. 2A and Fig. S2A and D). Once reaching the expert level, mice were implanted with a 64-channel tetrode probe in both dorsal CA1 and RSC (Fig. S2B, see Materials and Methods). The implantation did not affect the performance of mice after the recovery (Fig. S2C). Neuronal ensemble spiking activities were recorded during DSAT and the sleep phase (Fig. 2A). Based on the theta-delta ratio in CA1 and electromyographic (EMG) signal, we identified 3 brain states: wake, NREM, and REM sleep (Fig. 2B, see Materials and Methods). REM sleep and wake state had a high theta-delta ratio (>2), while NREM sleep displayed a strong delta power with a low theta-delta ratio. The EMG signal was low in REM sleep and NREM sleep due to the resting state. The local field potentials (LFPs) in CA1 and RSC were tightly synchronized in these 3 brain states (Fig. S2E). There were strong theta rhythm correlations in REM sleep and the waking state, while the correlation in the spindle rhythm of both areas was high in NREM sleep. During the delay period or food consumption behavior in DSAT, the correlation matrix of LFPs showed evident synchronization between CA1 and RSC in delta and theta oscillations.

Next, we focused on the neural activity occurring during hippocampal SWRs when the CA1 and RSC exhibited high-frequency oscillations (140 to 250 Hz, ripple and ripple-like, respectively) during the task and sleep phase. Temporal analysis of the correlation between hippocampal SWRs and RSC ripple-like events indicated that most RSC ripple-like events occurred concurrently with hippocampal SWRs (Fig. 2C). Moreover, hippocampal ripple power was positively correlated with RSC ripple-like power during the task (*r* = 0.195, *P* = 6.727 × 10^−5^) (Fig. 2D). This coordination between CA1 and RSC was also observed during sleep, showing comparably event power (Fig. 2D, *r* = 0.364, *P* = 1.436 × 10^−180^). However, the RSC oscillations around the time of hippocampal SWRs during sleep exhibited a larger power increase compared to the task phase (Fig. 2E and F), with a marked increase around the onset of SWRs and a substantial decrease after SWRs (Fig. S2F and G), reflecting strongly coordinated activity between the hippocampal SWRs and RSC oscillations during sleep.

The wavelet power coherence between hippocampal SWRs and RSC ripple-likes revealed a peak in the ripple rhythm (Fig. S2H and I). The mean peri-SWR spectrogram showed an increased power in RSC during the task phase, but compared to the task phase, the sleep phase exhibited a more pronounced power increase around the onset of SWRs and a shift of RSC oscillation power across the slow-to-fast oscillation bands (50 to 300 Hz) from pre-SWR to post-SWR (Fig. 2G), the shift of which may reflect the hippocampal SWR–RSC interaction [31]. This shift was not observed around SWR during the spatial task (Fig. 2G). Importantly, chemogenetic inhibition in the RSC during the sleep phase revealed a critical impact on the neural oscillatory interaction within the CA1 and RSC regions. Compared to vehicle treatment, administration of CNO led to notable changes in the power spectral density, most prominently in the ripple band in the CA1 and ripple-like band in the RSC (Fig. S3A and B). This was further substantiated by the reduction in the duration of ripples observed in the CA1 region under CNO influence in RSC, as well as a marked decrease in ripple rate and peak normalized power in both CA1 and RSC (Fig. S3C to E). These alterations underscore the causal link of RSC and CA1 on the dynamics of ripple events during sleep. Moreover, a greater proportion of RSC cells exhibited obvious increases in firing frequency around the SWRs during sleep than during the task phase (Fig. 2A, H, and I). These findings suggested strongly coordinated activity between the hippocampal SWRs and RSC oscillations during sleep, indicating that sleep is a crucial phase for memory consolidation in the hippocampus and RSC.

### CA1–RSC reactivation within hippocampal SWRs

**Fig. 3.**
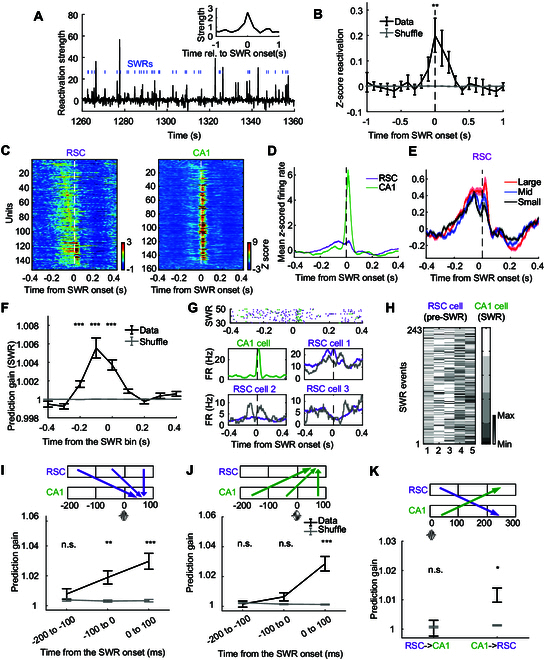
Reactivation dynamics between hippocampus and RSC during SWRs, and hippocampus–RSC interaction around SWRs during sleep. (A) Population reactivation of a locomotion pattern during an example sleep session, with SWR instances highlighted in blue. Inset shows a histogram triggered by SWR-related reactivations. (B) Mean *z*-scored reactivation histogram across sleep phases (*n* = 13 sessions). Gray trace showed the result for the same data when SWR timings were randomly shuffled, *P* = 0.0085, at lag 0, one-sided Wilcoxon signed rank test. (C) Left: *Z*-scored PSTHs of all modulated RSC neurons in relation to SWR onset, sorted by the timing of peak firing in a ±300-ms window. Right: Same as left but for all modulated CA1 neurons. (D) Mean *z*-scored PSTH across all modulated neurons in CA1 (green) and RSC (violet). All shaded areas within this study indicates SEM. (E) Mean *z*-scored SWR-correlated PSTH for all modulated RSC neurons (*n* = 200 units), categorized by the sizes of SWR events during sleep (red for large, blue for medium, black for small events). (F) Prediction gain of SWR occurrence. Each column at bin t depicts the prediction gain of SWR occurrence at time 0 from predictor RSC patterns at bin t. Gray are shuffled data, in which the RSC patterns were shuffled relative to SWR times. (G) Activity of sample RSC–CA1 neuron pairs around SWR onsets. Top: Combined spike raster plots for a simultaneously recorded CA1 neuron (green) and RSC neuron (violet) around SWR onset. Bottom: PSTHs for the same CA1 (green) and RSC (violet) neurons, categorized by the spike count of the CA1 neuron (≥1 spike shown in violet/green; 0 spikes in gray). (H) Example dataset used for GLM prediction analysis. Matrix denotes spike counts in the pre-SWR time window (−100 to 0 ms relative to SWR onset) of 5 RSC cells. The right shows spike counts in the subsequent SWR time window (0 to 100 ms) of one CA1 cell. Spike counts of the CA1 cell sort spike counts of all cells. Data corresponding to the data in (H): the first, second, and third columns of the RSC matrix correspond to “RSC cell 1”, “RSC cell 2”, and “RSC cell 3”, respectively. (I) Prediction of CA1 single-cell spiking during SWRs from ensemble spiking patterns in RSC across varying time windows (*n* = 76 predicted CA1 cells). Black error bars indicate mean ± SEM. Prediction gain for actual data. Gray error bars indicate mean ± SEM. Prediction gain for shuffled data. Columns represent varying time windows used as predictor data. CA1 spiking within SWRs (0 to 100 ms) could be predicted significantly better than shuffled data from RSC ensemble spiking patterns from −100-ms to 0-ms window (*z* = 2.983, *P* = 0.0026) and 0- to 100-ms window (*z* = 3.748, *P* = 1.780 × 10^−4^) but not from the −200- to −100-ms window (*z* = 1.220, *P* = 0.223). (J) Prediction of RSC single-cell spiking during SWRs from ensemble spiking patterns in CA1 (*n* = 100 predicted RSC cells). RSC spiking within SWRs could not be significantly predicted from pre-SWR CA1 ensemble spiking patterns but could be predicted from CA1 spiking within SWRs (−200 ms to −100 ms: *z* = −1.360, *P* = 0.174; −100 ms to 0 ms: *z* = −0.230, *P* = 0.818; 0 to 100 ms: *z* = 4.445, *P* = 8.792 × 10^−6^). (K) Prediction of post-SWR time window from ensemble spiking patterns within SWRs (0 to 100 ms). RSC ensemble spiking patterns within SWRs could not forecast CA1 firing in the post-SWR window (200 to 300 ms; left; *n* = 76 predicted CA1 cells, *z* = −0.576, *P* = 0.565), but CA1 ensemble spiking patterns within SWRs significantly predicted RSC firing in the post-SWR window (200 to 300 ms; right; *n* = 100 predicted RSC cells, *z* = 1.976, *P* = 0.0482). *P < 0.05, **P < 0.01, ***P < 0.001 for GLM prediction values, 2-tailed rank-sum test compared to shuffle.

To explore whether RSC firing around SWRs during sleep represent reactivation of awake experiences, we employed a previously developed reactivation strength analysis [10] (Fig. S4, see Materials and Methods). It used a template during the reference epoch (task phase) to track the time-resolved reactivation of recorded neuronal ensembles during the match epoch (sleep phase), which generated a reactivation strength line, and thus correlated the instantaneous reactivation with SWRs (Fig. 3A). CA1–RSC pairwise correlation patterns were defined as reference templates when mice ran in the stem and left/right side arms of the maze, during which mice departed from the start area, made a decision, and approached the reward area in the task. The CA1–RSC reactivation strength was significantly enhanced at the time of SWRs (*P* = 0.0085, at lag 0, one-sided Wilcoxon signed rank test; Fig. 3B), suggesting that CA1–RSC associations observed in decision-making during DSAT were reactivated during sleep, which specifically emerged around SWRs during NREM phase.

### CA1–RSC dialogue around hippocampal SWRs

It is not clearly known how RSC cooperates with hippocampal cells or SWRs during sleep. To examine this, we generated the ±1-s peri-SWR time histograms (PSTHs) by aligning the spiking activity of all cells to the onset of SWRs and then normalized the activity to baseline (from −1 to −0.5 s) (Fig. S5). A fraction of RSC cells (152/200, 76%) and CA1 cells (161/190, 84.74%) were significantly modulated around SWRs (Fig. 3C). Notably, most RSC cells started firing before the onset of SWRs, while CA1 cells preferred to fire after SWR onsets (Fig. 3C and D). Furthermore, SWR events were divided into 3 categories as large, middle, and small, according to the ripple amplitude [31]. Distinct spectral LFP patterns were observed in each SWR subtype (Fig. S6A), with significant differences in their spindle and high-frequency bands around the SWRs (Fig. S6B). Besides, the peri-SWR spiking of RSC neurons was proportional to SWR size [−100 ms to 0 ms: *F*(2,453) = 9.111, *P* = 1.320 × 10^−4^; 0 to 50 ms: *F*(2,453) = 6.912, *P* = 0.0011, one-way ANOVA] (Fig. 3E and Fig. S6C). These results implied the information transmission around the SWRs during sleep.

Based on analysis with cross-validated generalized linear models (GLMs), the occurrence of hippocampal SWRs could be significantly predicted by RSC ensemble activities around the SWRs (Fig. 3F), particularly in the time window of −100 to 0 ms to SWR onset (*P* = 3.052 × 10^−5^, 2-tailed Wilcoxon signed rank test). Furthermore, to explore whether RSC pre-SWR ensemble firing patterns were relevant to the subsequent hippocampal neuron activities, we employed the GLMs to predict the spike number of individual CA1 neurons within SWRs (0 to 100 ms). The pre-SWR firings of some RSC cells were correlated with the CA1 cells, either positively or negatively (Fig. 3G and H). Moreover, we investigated whether hippocampal activity influenced RSC during and around SWR events. It was found that within SWRs, the firing patterns of CA1 and RSC were reciprocally predictive (Fig. 3I and J). During the pre-SWR periods, the firing activity of RSC reliably predicted the subsequent CA1 activity (Fig. 3I), whereas the prediction gain of CA1 activity for RSC was not significantly better than chance during the same pre-SWR periods (Fig. 3J). Contrarily, CA1 ensemble activities within SWRs predicted RSC spiking in the post-SWR window (200 to 300 ms), but the RSC patterns within SWRs did not significantly predict post-SWR CA1 spiking (Fig. 3K). In the post-SWR, the firing rate of RSC neurons was correlated with SWR sizes [Fig. 3E, from 200 to 300 ms, *F*(2,453) = 11.577, *P* = 1.249 × 10^−5^, one-way ANOVA], suggesting that RSC activity was modulated by CA1 neurons around SWRs. Similar predictions had been detected from the 100- to 200-ms window (Fig. S7A and B), as well as for pre-SWR windows in the 0- to 200-ms period (Fig. S7C and D). These patterns point to a directional flow of information from RSC to the hippocampus and back to the RSC surrounding SWRs during sleep, implying an intricate process of memory consolidation that involves coordinated hippocampal–cortical interactions.

### Enhanced CA1–RSC dialogue after spatial training

**Fig. 4.**
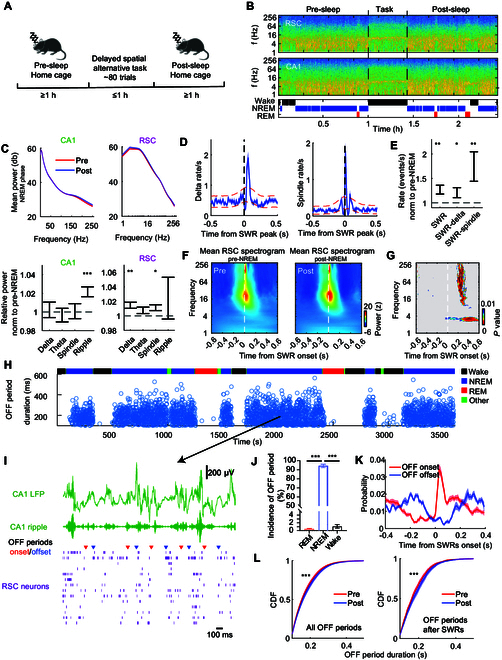
Awake experiences changed RSC activities during NREM sleep. (A) Experimental schedule. The spatial task phase was flanked by 2 sleep phases (pre-sleep and post-sleep). (B) RSC (top) and CA1 (middle) spectrograms and hypnogram in brain states (wake, NREM, or REM sleep) for an example session (simultaneous RSC and hippocampus recordings) including pre-sleep, spatial task, and post-sleep phases. Spectrogram colors represent power from low (blue) to high (yellow). (C) Top: Average power spectral densities (*n* = 27 sessions) during NREM in CA1 (left) and RSC (right). Bottom: Ratios of post-NREM to pre-NREM average power spectral density in delta (1 to 4 Hz), theta (6 to 10 Hz), spindle (10 to 20 Hz), and ripple (140 to 250 Hz) bands in CA1 (left) and RSC (right). *P < 0.05, **P < 0.01, ***P < 0.01, Wilcoxon signed rank test. (D) Left: Average cross-correlogram between CA1 SWR peaks and RSC delta wave peaks. Right: Average cross-correlogram between CA1 SWR peaks and RSC spindle peaks. Red lines, 95% confidence intervals. (E) Incidence of post-NREM SWR, SWR–delta, and SWR–spindle normalized to the incidence in pre-NREM. *P < 0.05, **P < 0.01, ***P < 0.001, Wilcoxon signed rank test. (F) Mean spectrogram from RSC locked to the onset of CA1 SWRs (left, pre-NREM; right, post-NREM). (G) Difference in the power of each frequency band at each lag around SWR onset between the pre-NREM and post-NREM in RSC. One-way ANOVA, *P* < 0.01. Nonsignificant (*P* > 0.01) areas are shown in gray. (H) Top: Hypnogram for an example sleep phase. Bottom: Dynamics (occurrence and duration) of OFF periods during different vigilance states. (I) Representatives of simultaneously recorded CA1 oscillations (green) and spikes of RSC neurons (bottom, violet). RSC neurons fired in clusters with an “ON-OFF” cyclic pattern: red triangles indicate OFF period onsets; blue triangles indicate OFF period offsets. (J) Incidence and average duration of OFF periods according to the behavioral states. (K) Mean probability of RSC OFF period onsets (red) and offsets (blue) in relation to CA1 SWR onsets. (L) Cumulative distribution function (CDF) of the durations of all the OFF periods (left) and the OFF periods that emerged 400 ms after SWRs (right). ***P < 0.001, Wilcoxon signed rank test.

RSC neural activities were correlated to hippocampal SWRs during sleep, so we hypothesized that waking experiences impacted CA1–RSC coordination during sleep, which could further support the theory that hippocampal–cortical dialogue is associated with memory consolidation after spatial learning. To verify this, we recorded 2 sleep periods (pre-sleep and post-sleep) flanking the task period (Fig. 4A) and hypothesized that CA1–RSC communication during NREM was strengthened after the spatial navigation task. First, the power of oscillations was analyzed during pre-NREM and post-NREM in CA1 and RSC (Fig. 4B). A notable increase in ripple power was observed during post-NREM compared with pre-NREM in the power spectrum of the CA1. In contrast, delta and spindle power in RSC was significantly higher during post-NREM than during pre-NREM (Fig. 4C). There is no significant difference in oscillations between post-sleep and pre-sleep during REM phase (Fig. S8), highlighting the more critical role of NREM sleep in memory consolidation in this task. Delta and spindle waves were dominant in time close to SWRs (Fig. 4D), with rates peaking at ~60 ms and ~10 ms after SWR, respectively, indicating that delta and spindle waves in RSC generally followed SWRs. The occurrences of SWRs, SWR–delta, and SWR–spindle sequences rose after training (Fig. 4E), implying enhanced hippocampal–cortical dialogue in memory consolidation [5,13]. Although spectral LFP patterns during pre-/post-NREM both showed high power around SWRs, there were significant differences after SWRs (Fig. 4F and G). We hypothesized that these changes resulted from the spiking silence, as delta waves reflected the cortical down states when cortical neurons stopped firing [1]. The OFF periods were identified when at least 5 RSC neurons were silent for at least 100 ms [31] (Fig. 4H and I). OFF periods predominantly occurred during NREM but not during REM sleep or wake state (Fig. 4J). Our analysis showed not only a high probability of RSC ensemble activity termination immediately after SWRs (Fig. 4K) but also a growth in the duration of OFF periods during post-NREM compared with pre-NREM, especially the OFF periods that emerged after SWRs (Fig. 4L).

### Biased modulation of RSC activity around hippocampal SWRs after spatial training

**Fig. 5.**
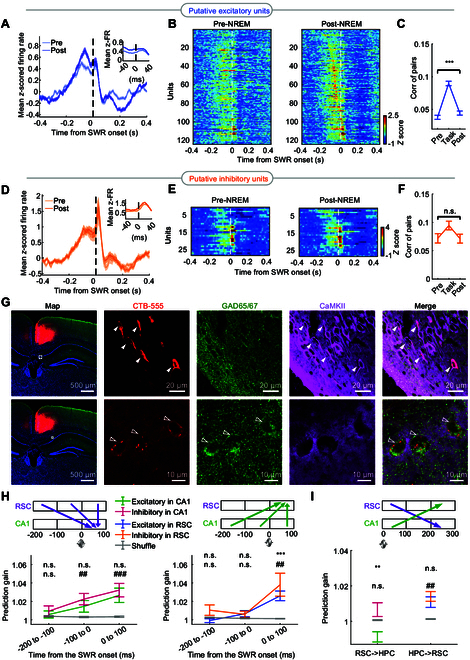
Cell type analysis in the RSC and anatomical/functional connectivity between CA1 and RSC. (A) Mean *z*-scored PSTH of all modulated RSC putative excitatory neurons (*n* = 158 units) relative to SWR onsets, contrasting pre-NREM (light blue) and post-NREM (dark blue) phases. (B) *Z*-scored SWR-related spiking heatmaps for modulated putative excitatory RSC neurons during pre-NREM (left) and post-NREM (right) sleep, sorted by post-NREM peak firing timing within a ±300-ms window. (C) Correlation plots for all CA1–RSC excitatory neuron pairs (*n* = 273) with significant positive correlations during running (refer to Fig. S11B and E), shown across pre-NREM, task (in the spatial task), and post-NREM phases. (D) Analysis similar to (A), for all putative inhibitory neurons (*n* = 42 units) of RSC, with correlations depicted for pre-NREM (light orange) and post-NREM (dark orange) sleep. (E) Replication of analysis in (B) for putative inhibitory neurons of RSC. (F) Correlation analysis for all CA1–RSC inhibitory neuron pairs (*n* = 79). (G) Coronal section showing CTB injection in the RSC (left, Map) and zoom-in image of the specific area (white square in Map) for retrograde labeling of CTB in the CA1 (right). The zoom-in images show CTB overlap with CaMKII in rostral medial CA1 (top panels, white arrows) and GAD65/67 in the stratum oriens of CA1 (bottom panels, hollow arrows). Red, CTB; green, GAD65/67 immunostaining; purple, CaMKII immunostaining; blue, DAPI staining; Merge, combined CTB, GAD65/67, and CaMKII. Scale bars, Map: 500 μm, zoom-in images: 20 μm (top) and 10 μm (bottom). (H) Left: GLM-based prediction of spiking in CA1 putative excitatory and inhibitory neurons during SWRs from RSC ensemble spiking patterns across varied time windows (*n* = 34 predicted CA1 excitatory cells, *n* = 42 predicted CA1 inhibitory cells). Green and red error bars represent mean ± SEM for actual data for excitatory and inhibitory neurons, respectively. Gray bars indicate mean ± SEM for shuffled data. Right: Prediction of spiking in RSC putative excitatory and inhibitory neurons during SWRs from CA1 ensemble patterns (*n* = 80 predicted RSC excitatory cells, *n* = 20 predicted RSC inhibitory cells). Blue and orange bars represent mean ± SEM for RSC excitatory and inhibitory neurons, respectively. **P < 0.01, ***P < 0.001 for predicted putative excitatory neurons, ^##^*P* < 0.01, ^###^*P* < 0.001 for predicted putative inhibitory neurons, 2-tailed rank-sum test compared to shuffle. (I) GLM-based prediction of post-SWR spiking from intra-SWR ensemble patterns. Left: Predictions for CA1 putative excitatory and inhibitory neurons (*n* = 34 excitatory, *n* = 42 inhibitory). Right: Predictions for RSC putative excitatory and inhibitory neurons (*n* = 80 excitatory, *n* = 20 inhibitory) during the post-SWR window. **P < 0.01, ***P < 0.001 for predicted putative excitatory neurons, ^##^*P* < 0.01, ^###^*P* < 0.001 for predicted putative inhibitory neurons, 2-tailed rank-sum test compared to shuffle.

We then wondered whether individual RSC neurons were involved in memory consolidation and whether different types of neurons played the same role in this processing. To determine the firing characteristics of RSC neurons across the 3 recording phases (Fig. 4A), the same units were tracked during these periods (Fig. S9, see Materials and Methods). Then, RSC putative excitatory and inhibitory neurons were identified by using principal components analysis (PCA) [31] (Fig. S10A and B). Meanwhile, CA1 neurons were classified based on the waveform and firing features (Fig. S10C and D), according to the reported article [37]. RSC neurons in NREM sleep displayed the lowest firing rate in comparison to REM sleep and wake state (Fig. S10E). Furthermore, the PSTHs of RSC neurons were compared between the pre-NREM and post-NREM. The firing rate of RSC excitatory neurons increased significantly in the pre-SWR window (averaged from −100 to 0 ms: *z* = 5.429, *P* = 5.676 × 10^−8^, 2-tailed signed rank test compared to pre-NREM), while RSC excitatory ensemble spiking patterns displayed a prominent inhibition in the post-SWR window (averaged from 200 to 300 ms: *z* = −6.015, *P* = 1.802 × 10^−9^) (Fig. 5A and B), which were critical periods for CA1–RSC information interaction (Fig. 3). By contrast, these changed firing features around the SWRs were not present in RSC inhibitory neurons (−100 to 0 ms: *z* = 1.230, *P* = 0.219; 200 to 300 ms: *z* = −1.230, *P* = 0.279) (Fig. 5D and E).

Besides, the correlations of CA1–RSC cell pairs were higher during post-NREM than during pre-NREM (Fig. S11A), revealing that the patterns of co-firing between CA1–RSC pairs formed during wakefulness were reactivated during sleep in line with the consolidation theory [2,6,11]. To further examined how reactivations across the CA1–RSC network were linked to awake experiences, the same awake CA1–RSC pairwise correlations in the trajectory of approaching the reward after the delay were defined as Task correlations (Fig. S11B) as in the reactivation strength analysis (Fig. 3A and B). Substantial CA1–RSC pairs in the trajectory showed significantly positively correlated spike trains (352 of 1,007; 34.96%) (Fig. S11C to E). A small fraction (86 of 1,007; 8.54%) was significantly negatively correlated, while most of the remaining (569 of 1,007; 56.50%) was unreliably correlated (Fig. S11E and F). We found that for RSC excitatory neurons, but not for RSC inhibitory neurons, correlations of significantly positively correlated CA1–RSC pairs exhibited a noteworthy increase from pre-NREM to post-NREM (Fig. 5C and F). The rise in co-activity was considerably associated with co-activity in the Task trajectory for CA1–RSC excitatory pairs (Fig. S11E and F).

To further identify the contributions of excitatory and inhibitory neurons to the interaction between the CA1 and RSC, we injected the retrograde tracer CTB-555 into RSC to trace the monosynaptic projection inputs from CA1 (Fig. 5G). The CTB-labeled neurons in the CA1 were precisely colocalized with markers CaMKII and GAD65/67, indicating the presence of both excitatory and inhibitory neurons in CA1 projecting to the RSC (Fig. 5G). These results suggested a structural basis for CA1–RSC connectivity to play a role in physiological processes associated with behavioral functions. By using the GLM analysis, the activities of excitatory and inhibitory neurons were mutually predictive for the SWR events (Fig. 5H). The firing activity of RSC significantly predicted the inhibitory neurons in the CA1, but not excitatory neurons, during the pre-SWR periods (Fig. 5H). In the post-SWR phase, the CA1 activity emerged as a significant predictor of RSC inhibitory spiking, whereas the prediction gain for CA1 neurons either was not significant or fell below shuffled baselines (Fig. 5I). These asymmetric predictions validate the directional exchange of information within the circuit of RSC–CA1–RSC surrounding SWRs and further suggest that the observed suppression of excitatory neurons in the RSC after SWR onsets may be related to both CA1 inhibitory neurons and local RSC inhibitions.

### Differences in hippocampal SWR-correlated firing patterns of RSC neurons encoding divergent spatial information

**Fig. 6.**
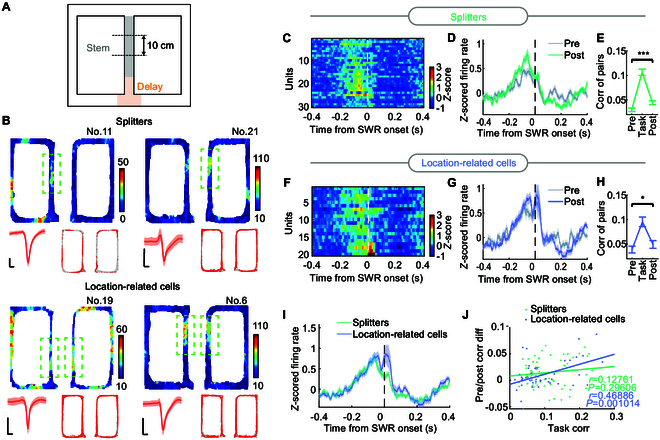
RSC excitatory neurons, which distinguished future trajectories fired preceding SWRs. (A) Schematic of the figure-eight maze indicating the stem (gray shaded area, the periods after the delay, and before the turning) used in the seeking and discrimination of splitters and location-related cells. (B) Four examples of RSC putative excitatory neurons (top, splitters; bottom, location-related cells). Firing heatmaps are shown for the task phase. Color bars are in hertz. The notable firing in the stem area is delineated by a dashed green box. The mean waveform of one neuron is shown in the bottom left of the subgraph; the bars are 200 μs and 50 μV. Trajectory (gray line) with superimposed spike locations (red dots) in the bottom right of the subgraph. (C) *Z*-scored SWR-related spiking heatmaps of splitters in post-NREM sleep, sorted by the timing of post-NREM peak firing in a ±300-ms window. (D) Mean *z*-scored PSTH of splitters (*n* = 30 units) in relation to SWR onsets during pre-NREM (gray) and post-NREM. (E) The correlations of CA1–RSC splitter pairs that show significant positive correlations while running are shown for pre-NREM, task, and post-NREM phases. (F) Same as (C) but for RSC location-related cells. (G) Same as (D) but for RSC location-related cells (*n* = 20 units). (H) Same as (E) but for CA1–RSC location-related cell pairs. (I) Mean *z*-scored PSTH of splitters (green) and location-related cells (blue) in relation to SWR onsets in post-NREM sleep. (J) Pearson’s correlation of CA1–RSC pair correlations. Green and blue correspond to CA1–RSC splitter pairs and CA1–RSC location-related cell pairs, respectively.

The representations of SWR-modulated RSC neurons in the awake state still remained unclear, so we further explored the activity patterns of RSC excitatory neurons that encoded spatial information during the task phase. Since RSC neurons are thought to anticipate and differentiate between trajectories leading to reward locations on the left or right sides [24,25], the maze stem region was divided into 3 subareas (each 10 cm) to investigate the role of RSC in different navigation period (Fig. 6A). Two types of RSC cells encoding spatial information in the stem-arm were identified (see Materials and Methods). One group consisted of splitter cells [24,38,39], which are neurons that differentiate between future routes by firing differentially depending on whether the trial is a left-turn or right-turn trial (Fig. 6B, top). These cells are named splitters because they “split” or distinguish overlapping spatial trajectories at shared segments of the maze [40,41]. The second group resembled traditional location-related cells, which showed parallel firing rates in both left-turn and right-turn trials (Fig. 6B, bottom).

Interestingly, splitters tended to reach peak firing rates before SWRs (Fig. 6C). In contrast, some of the location-related cells not only elevated firing rates in pre-SWR but also reached higher firing rates within SWRs (Fig. 6F). There were significant differences in firing frequency between the 2 clusters within SWRs (averaged from 0 to 100 ms, *z* = 2.452, *P* = 0.0142, 2-tailed rank-sum test; Fig. 6I). Comparing the firing patterns during pre-NREM and post-NREM, both clusters showed obviously biased modulated PSTHs around SWRs (Fig. 6D and G) and considerably strengthened correlation of CA1–RSC pairs (Fig. 6E and H). Our analysis revealed that for RSC location-related cells, but not for splitters, the rise in correlations of CA1–RSC pairs between pre-NREM and post-NREM was positively correlated with coactivity in the Task route (Fig. 6J). These observed phenomena indicate that RSC neurons, encoding various types of spatial information, are selectively modulated by the hippocampal SWRs.

## Discussion

Our results showed that bilateral lesions and silencing of RSC neurons impaired behavioral performance in DSAT, particularly affecting the process of memory consolidation during sleep (Fig. 1). We then reported hippocampal CA1–RSC coordination in distinct time windows around SWRs in the states of sleep and wakefulness, especially in peri-SWRs during sleep (Fig. 2). The joint CA1–RSC neuronal representations established during the spatial task were reactivated during SWRs in the following NREM phase (Fig. 3B). Numbers of RSC neurons (152/200, 76%) were significantly tuned around the SWRs (Fig. 3C). Patterned activation in the RSC ensemble preceded and predicted the subsequent hippocampal activity during SWRs. In contrast, hippocampal patterns during SWRs overshadowed the subsequent RSC activity. Altogether, the findings revealed an RSC–CA1–RSC circuit of information flow around the onset time of SWRs during sleep (Fig. 3). Moreover, awake experiences in DSAT resulted in increased pre-SWR activities of RSC excitatory neurons and longer overshadowing periods in the RSC population (Figs. 4 and 5), which suggested their roles in spatial memory consolidation during sleep after DSAT. The demonstrations of monosynaptic projections and GLM analysis revealed that RSC activity significantly predicted the firing of CA1 inhibitory neurons, which may subsequently influence the suppression of excitatory neurons in the RSC (Fig. 5). Finally, we found that 2 clusters of RSC excitatory and inhibitory neurons encoding divergent spatial information were distinctly modulated by SWRs during sleep (Fig. 6). These results suggested that spatial information encoded by RSC was transferred to CA1 during sleep and CA1 selectively sent feedback to distinct RSC neurons as a modulatory instruction, which occurred around the crucial period of SWRs to facilitate memory consolidation.

### Critical role of RSC in spatial memory consolidation

Although behavioral findings have shown RSC lesion-induced deficits in spatial tasks [25,42], we found that RSC was not required for learning the spatial task by using either lesion (Fig. 1E) or chemogenetic inhibition (Fig. 1H). One possible explanation is compensation across other brain structures mediated by parallel interconnections after extensive forced training [16]. Our study showed that lesions and silencing of RSC neurons significantly disrupted behavioral performance in retrieving spatial memory (Fig. 1F) and memory consolidation during sleep (Fig. 1I), consistent with the role of long-lasting memory storage in RSC after learning as shown in other studies [25,43,44]. These results indicated that RSC played a crucial role in spatial memory storage and recall. Given the functional similarities and anatomical connectivity with the hippocampus [16,44], memory consolidation in RSC may be relevant to the communication between the hippocampus and RSC [44,45].

### Interplay of RSC and hippocampal oscillations

The relationship between RSC oscillations and CA1 SWR events during NREM appeared remarkably strong (Fig. 2). The shifts in RSC oscillations from pre-SWR to post-SWR during sleep suggested information processing via interaction between the 2 regions (Fig. 2F and G), consistent with previous studies [31]. Notably, the chemogenetic inhibition of RSC significantly affected the SWRs in the CA1 and ripple-like band in the RSC, suggesting that the RSC and CA1 interaction, particularly during ripple events in sleep, represents a key aspect of how the brain integrates and stabilizes memories (Fig. S3A to E). Although research on cortical ripples, particularly ripples in the RSC, started later compared to well-studied hippocampal SWRs, extensive findings have emerged in recent studies, offering substantial support and basis for our study. Khodagholy et al. [34] first revealed high-frequency ripple oscillations in the rat association cortex, particularly noting the coupling of these ripples in the RSC with hippocampal ripples. The retrosplenial neurons phase-locked to local theta and gamma rhythms during hippocampal SWRs in offline states [29,35,46]. This interplay between RSC and hippocampal oscillations is important in spatial and episodic memory processing, memory consolidation, and information transfer [31,35,47,48], consistent with our findings.

### RSC–CA1 interaction and SWR-associated activity

We also found that neuronal activity in a substantial fraction of RSC neurons (76%) was associated with the CA1 SWR events, and they were characterized by a tendency to increase firing frequency before the onset of SWR events and decrease it ~50 ms after the SWRs (Fig. 3C and D). Similar patterns have been observed in the anterior cingulate cortex [49]. In addition, the RSC ensemble patterns during pre-SWR (from −100 to 0 ms) predicted the occurrence of SWR events and the firing count of CA1 neurons within SWRs (0 to 100 ms) (Fig. 3I), suggesting that RSC played a role in modulating SWRs, such as SWR-correlated reactivation contents [10,50]. Accordingly, the population activity of CA1 within SWRs predicted the RSC firing rates after SWRs (200 to 300 ms) (Fig. 3K), indicating that hippocampal replay within SWRs tuned RSC activity. These findings exhibited an RSC–hippocampus–RSC information flow around SWRs during sleep, implying a fine-grained memory consolidation mechanism via hippocampal–cortical coordination. Recent articles focused on the interaction between the RSC and the hippocampus in REM sleep with Granger causality analysis in field potentials. These articles found more robust information transmission from the hippocampus to the RSC in theta rhythm of REM sleep [28,30] and fluctuations in interaction strength in hippocampus and RSC during phasic REM, which played a functional role in hippocampal–cortical memory consolidation [30]. Here, we found a cortical–hippocampal–cortical information flow during NREM, similar to the former report in the auditory cortex (AC) and hippocampus [10]. However, the firing pattern of AC studied previously differed from that of the RSC. A small proportion (36%) of AC neurons were modulated by SWRs [10], while we found that neuronal firings in most of the recorded RSC cells (76%) were associated with SWRs (Fig. 3C), which was prompted by the functional and anatomical connections of the RSC with the hippocampus [26,27,51]. Moreover, AC neurons had an ensemble that fired before SWRs and a subpopulation that fired after SWRs, while most neurons in RSC enhanced firing before SWRs and reduced firing rate after SWRs. This discrepancy between AC and RSC implies a diversity of information interaction between the hippocampus and other structures. Meanwhile, we found that the joint CA1–RSC neural activity relevant to decision-making during waking experience was reactivated with the greatest strength when SWRs occurred, corresponding to previous findings that the hippocampus–amygdala pathways reactivated fear memories near SWRs [14].

### Hippocampal–RSC dialogue in spatial encoding

In our study, we observed that RSC excitatory and inhibitory neurons decreased their firing rates during NREM relative to the awake state (Fig. S10E), consistent with reported findings [28]. These findings may be attributed to the up-down transition in the cortex during NREM, as indicated by extended OFF periods post-NREM (Fig. 4L). Interestingly, the joint hippocampal and cortical activities heightened after training (Fig. 4), implying a strengthened role in memory consolidation. Although the RSC is not involved in generating CA1 SWRs anatomically and functionally [4,52], the RSC can send output to CA1 indirectly via the entorhinal cortex and subiculum [53,54]. Our electrophysiological results do not exclude the possibility of such indirect projections from the RSC to CA1. Additionally, several RSC putative excitatory neurons recorded also discharged with increased frequency after SWRs, which tended to encode location information in the spatial task. Moreover, neurons with predictive decision-making capability [24,25,39,40] tended to discharge before SWRs (Fig. 6). These results indicate that neurons encoding different information may be biased by the hippocampus, similar to the report on the hippocampus and nucleus accumbens network [55]. As RSC plays a critical role in encoding and storing memories [19,21,56–58], we speculate that this is the reason for the specific changes in RSC firing pattern modulation by hippocampal SWRs after daily training.

### Modulation of excitatory and inhibitory neurons in the RSC by hippocampal activity

Our study revealed that RSC excitatory neurons were recruited at a higher frequency before SWRs (specifically in −100 to 0 ms) in post-NREM compared with pre-NREM (Fig. 5A) when RSC ensembles predicted hippocampal activity during subsequent SWRs (0 to 100 ms) (Figs. 3I and 5H), driving hippocampal replay of specific memories, which is consistent with the theory of targeted memory reactivation [50]. Conversely, RSC inhibitory neurons displayed an increased activation after the onset of SWRs (Fig. 5D) and the extended OFF period duration in RSC (Fig. 4L). This observation resonates with a recent optogenetic study, suggesting that hippocampal CA1 may modulate RSC in supporting memory consolidation by activating RSC inhibitory neurons [31]. Importantly, we observed that there is no significant increase in activation of RSC inhibitory neurons within SWRs in post-NREM compared to pre-NREM (Fig. 5D). This conclusion may be limited by our recording from only a small number of inhibitory neurons. However, we showed that the firing of CA1 inhibitory neurons has been predicted by RSC activity during pre-SWR periods, and then predicted both excitatory and inhibitory neurons in the RSC in turn (Fig. 5H). We further found monosynaptic projections from CA1 inhibitory neurons to the RSC (Fig. 5G), consistent with inhibitory input from the CA1 region to the RSC [59–61] or CA1 indirect output to RSC through subiculum [15,46,61]. Therefore, excitatory neurons significantly decreased their firing rates after SWRs (200 to 300 ms) (Fig. 5A and B), which supported the possibility that the hippocampus interferes with or overshadows RSC for learning and memory processing [62].

## Materials and Methods

### Animals

Male C57BL/6J mice (8 to 12 weeks old, 24 to 32 g) from Shanghai Lingchang Biotechnology Company Limited were used in this study. The animals were maintained in a constant environment (temperature 21 ± 1 °C, humidity at 75 ± 3%) under a 12:12 h light–dark cycle (lights on at 07:00 AM). All experiments were conducted in accordance with the guidelines of the National Care and Use of Animals. All protocols were reviewed and approved by the Animal Care and Use Committee (the Department of Laboratory Animal Science) of Fudan University (no. 202011019S).

### Surgery

For the lesion experiment, 25 mice were anesthetized with isoflurane gas (induction: 4%; maintenance: 1 to 1.5%) and mounted on stereotaxic apparatus (RWD Instruments, Shenzhen, China). The skin was retracted, and holes were drilled through the skull above each of the injection sites [anterior-posterior (AP): −1.5 mm, dorsal-ventral (DV): 0.9 mm, medial-lateral (ML): ±0.3 mm; AP: −2.5 mm, DV: 1 mm, ML: ±0.3 mm; AP: −3.4 mm, DV: 1 mm, ML: ±0.5 mm]. Thirteen mice received RSC’s bilateral neurotoxic (NMDA, 0.09 M) lesions. NMDA was injected in volumes of 50 nl per site using a custom-made glass injection pipettes (100 μm diameter, about 20 to 30 μm in diameter at the tip) attached to Nanoject III (Drummond, catalog no. 3-000-207). The glass injection pipette was left in place 7 min after each infusion. An additional 12 mice received sham lesions of the RSC by injecting 0.9% saline.

For chemogenetic virus injection, adult C57BL/6J mice were anesthetized and mounted as lesion surgery described above. The skull was exposed, and holes were drilled above the injection sites (RSC: AP: −1.7 mm, DV: 0.9 mm, ML: ±0.4 mm). Mice were injected bilaterally with either experimental [AAV2/9-hSyn-hM4D(Gi)-mCherry] or control virus (AAV2/9-hSyn-mCherry) into the RSC. Virus (200 nl) was injected per site similar to the injection method described above. The glass pipette was left in place 10 min after the injection before retraction. Mice were recovered from the surgeries for about 2 weeks before subsequent behavioral training.

For electrophysiological probe implantation, mice were anesthetized with isoflurane (induction: 4%; maintenance: 1 to 1.5%). Electrophysiological signals were recorded from 5 mice using customized 64-channel tetrode arrays. Each array consisted of 8 shanks of tungsten microelectrodes (with 100- to 350-kΩ impedance; California Fine Wire, CFW-100-211) spacing 200 to 250 μm in a rectangular-shaped arrangement. Two sets of tetrodes (8 shanks each) were placed in the dorsal CA1 (AP: −1.5 to −2.5 mm; DV: 1.6 to 2 mm; ML: 1 to 2 mm) and RSC (AP: −1.3 to −1.8 mm; DV: 0.7 to 1 mm; ML: 0.1 to 0.5 mm) respectively. Two additional spherical electrodes (ST. STEEL 7 strand, A-M Systems, catalog no. 793200) were placed in the nuchal muscles to record EMG signals. A nichrome microwire (~5-kΩ impedance; California fine wire, CFW STABLOHM 675) was implanted in the ventral hippocampus as the reference for all channels. An additional screw was placed over the cerebellum and served as the ground. Arrays were anchored to the skull with Super Bond C&B using extra screws.

For the electrophysiological experiment combined with chemogenetics, we first injected chemogenetic virus as described above. After recovery for 2 weeks, mice were trained to do the DSAT. Customized 32-channel tetrode arrays were implanted in the dorsal CA1 and RSC as described above when mice reached the performance criteria.

For CTB retrograde tracing, adult C57BL/6J mice were injected unilaterally with 200 nl of CTB-555 (Invitrogen, catalog no. C34776). After 1 week, the mice were perfused for histology.

### Behavioral training and recordings

The behavioral apparatus was a PVC (polyvinyl chloride) figure-eight maze (78 cm long × 52 cm wide × 40 cm high) with extruding black walls and a white floor enclosed by gray curtains. Prior to training, mice were gradually water deprived to 85% to 90% of their baseline weight for about 5 d. They acclimated to the maze and received 20% sugar-water rewards (6 μl per trial) for 60 trials or 1 h per session. Except for recovery time, animals were hydrated in 1 to 2 g of HydroGel (99% H_2_O, ClearH20) per day to keep their body weight throughout the experiment. After water restriction and habituation, mice spent at least 5 sessions (60 trials or 1 h per session) of forced training on a shaping protocol. During this time, we unilaterally controlled the servo to gate correct decisions (forced choice trials). This stage was essential for training mice to alternate left and right directions without bias. Following forced training, mice performed tasks without assistance for at least 5 sessions, 60 trials, or 1 h per session for lesion experiments (80 trials or 1 h per session for neurophysiological recording experiments). When the mice chose the wrong direction, they were required to continue running along the return arm, re-enter the stem, and make the same correct choice on the subsequent trial. Once the performances reached 70% accuracy or at least 8 consecutive alternating trials for 2 continuing sessions, the mice were implanted with a probe. Animals were allowed to recover for at least 7 d with ad libitum food and water. After that period, the animals were connected to the recording cable for 2 h to adapt to the equipment. Electrophysiological recordings were performed using a multichannel acquisition processor (Cereplex Direct, Blackrock Microsystems, USA) sampled at 30 kHz. The headstage was connected to hydrogen balloons. In recording sessions, the behavioral phase was flanked by 2 sleep phases in the home cage (pre-sleep, post-sleep), each for at least 1 h. Electrophysiological recordings were performed separately in pre-/post-sleep and task sessions for easy storage and data alignment. Thus, we identified the same units in these 3 recording phases (see below). The animal’s position in the maze was captured using a camera (30 to 40 fps) mounted on the ceiling, and the recording videos were exported using a tracking system (Tracing Master V3.0, VanBi, China).

For chemogenetic mice training, after recovery from surgery and virus expression, mice were trained to do the task as described above. Activities of the RSC were then inhibited by intraperitoneal injection of CNO. Mice reaching above 75% accuracy in 7 consecutive sessions were defined as expert mice. Then mice were intraperitoneally injected with 2 mg/kg CNO (freebase) (HelloBio) 30 min before or immediately after the task.

### Preprocessing of electrophysiological signals

All analyses were performed using MATLAB R2021a (MathWork Inc., USA) custom-written scripts, buzcode-master package (https://buzsakilab.com/wp/resources/buzcode/), and CellExplorer-master package (https://cellexplorer.org).

Raw signals were first high-pass filtered 0.5 to 5 kHz and then thresholded to detect neuronal spikes. For 3 mice, detected spikes were semi-automatically sorted using “KlustaKwik2” (https://github.com/kwikteam/klustakwik2), followed by manual curation of the clusters using “Phy” (https://github.com/kwikteam/phy). Spike sorting was performed semi-automatically for 2 animals, using “Kilosort” (https://github.com/cortex-lab/KiloSort), followed by manual software “Phy”. Units were excluded if they had <900 spikes (minimum firing rate = 0.5 Hz), refractory period violation rates >0.01 (absolute refractory period of <1 ms), low amplitude (min amplitude >60 μV), obvious amplitude drift across the recording, and atypical waveforms (noise-like or only positive potentials).

Sleep stages were automatically scored by inspecting the hippocampus spectrogram and EMG signals, according to the scoring algorithm (https://github.com/buzsakilab/TheStateEditor). Briefly, signals were downsampled to 1,250 Hz to extract LFPs or EMG signals. EMG data were filtered (Butterworth band pass filter) between 20 and 150 Hz, smoothed in a gaussian window of ~50 ms, and *z*-scored to yield a signal EMG_z. The wake epochs in the home cage were marked if EMG_z passed the wake threshold. A channel from the hippocampus with the largest ripple (140 to 250 Hz) power was selected. The theta/delta ratio was calculated by the power of 6 to 10 Hz and 1 to 4 Hz in 256 samples and smoothed in a gaussian window of ~50 ms. Periods of NREM were associated with immobility (EMG_z < wake threshold) and low theta/delta ratio (<2). REM sleep was characterized by immobility and a high theta/delta ratio (≥2). The intervals (5 s) of 2 different stages were tagged as “other”. If the period of each stage was less than 30 s, this stage was marked as a transition stage (“other”).

Firing rate (FR) changes between wakefulness and NREM sleep were evaluated using the NREM /wake ratios, calculated as (NREM FR − wake FR)/(NREM FR + wake FR). Positive ratios indicated NREM FR > wake FR. REM /wake ratios were calculated in the same way.

### Offline detection of SWRs, delta waves, and spindles

We used the buzcode-master function to detect ripples. The LFP from the CA1 with the largest ripple power was filtered (140 to 250 Hz), squared, and normalized, yielding a signal **R**(**t**). Ripples were defined as events where R(t) remained above 1 for 15 to 200 ms, peaked at >4. Events separated by less than 20 ms were merged. We detected ripple-like events in RSC following the same procedure. Sharp waves were detected separately using LFPs from all CA1 channels, filtered with band-pass filter boundaries (5 to 40 Hz), and *z*-scored. LFP events between 20 and 400 ms exceeding 2.5 SD of the background signal were regarded as candidate sharp waves. SWR events were used for further analysis only if a ripple was simultaneously detected with a sharp wave. SWRs were detected only during NREM periods in the sleep session and during immobility (speed <3 cm/s) in the task session.

We used the FMAToolbox function (https://fmatoolbox.sourceforge.net/) to detect delta waves. The LFP recorded in the RSC with the largest ripple power was filtered (0 to 4 Hz) and *z*-scored, yielding *D*(*t*). We extracted time trains *t_onset_*, *t_peak_*, and *t_end_*, corresponding to the putative onset, peak, and end of delta waves, respectively. We discarded events lasting less than 150 ms or more than 450 ms. Delta waves corresponded to epochs where *D*(*t_peak_*) > 2 and *D*(*t_end_*) ≤ 0, or *D*(*t_peak_*) > 1 and *D*(*t_end_*) < −1.5.

For spindle detection, the recorded LFP in the RSC was band-pass filtered (10 to 20 Hz), squared, and *z*-scored, yielding *S*(*t*). Spindles were detected where *S*(*t*) remained above 2.5 for more than 500 ms and peaked at >5. We combined events separated by less than 400 ms and discarded merged events lasting more than 3 s.

SWR–delta (SWR–spindle) pairs corresponded to epochs where delta (spindle) peaks occurred after ripple peaks (0 to 250 ms).

### Spectral analysis

Spectrograms and power coherograms were constructed using either a short-time Fourier transform or a Morlet wavelet transform and were either plotted on a logarithmic scale or *z*-score normalized relative to a baseline period that was set as 0.5 to 1 s before the SWR peak.

### Reactivation analysis

Pairwise correlations for CA1–RSC pairs were calculated using the Pearson correlation coefficient on 50-ms-binned spike trains. The coefficients were separately calculated for pre-NREM, task running, and post-NREM periods.

To calculate the reactivation strength *R* in post-sleep epochs, we followed an approach described previously [14]. Based on this method, we derived CA1–RSC ensemble (at least 4 CA1 cells and 4 RSC cells) firing patterns during awake running epochs, employed those patterns as templates to match ensemble activities during identified sleep epochs, and quantified the similarity between the matched sleep network activity vectors and the awake template across time. Hippocampal CA1 and RSC cell spike trains while the animals were running in the stem and side arms before the reward areas (Fig. S11B) were binned (100-ms bins) and *z*-scored, yielding *Z*(*run*, *CA*1) and *Z*(*run*, *RSC*), the *n_bins_* × *n_cell_ z*-scored spike count metrics for CA1 and RSC. The CA1–RSC correlation matrix *C* for the running period (departure from the delay area and approach to the reward areas) was calculated as *C*(*run*) = *Z*(*run*, *CA*1)*Z*(*run*, *RSC*)*^T^*/*n_bins_* and defined as a template. The reactivation strength *R* was then calculated as *R*(*t*) = *Z*_*CA*1_(*t*)*C*(*run*)*Z_RSC_*(*t*)*^T^*, where *Z*_*CA*1_(*t*) and *Z_RSC_*(*t*) were the vectors of spike counts from CA1 and RSC neurons for the time bin *t* of post-sleep phase. The reactivation strength *R* over time was then *z*-scored before combining data across phases. The peri-SWR reactivation strength was calculated in a ±1-s window around ripple onsets (no inter-SWR minimum was applied). To assess the temporal relationship between reactivation and SWRs, we repeated the procedure 100 times by computing peri-SWR reactivation strength around random ripple onsets (the random amount of SWRs is consistent with the actual data). Finally, the mean peri-SWR reactivation strength *R* was calculated over all eligible post-sleep stages and compared the mean reactivation strength of the real versus shuffled data at the SWR onset using a Wilcoxon signed rank test.

### SWR-related spiking activity

For isolated units in pre-/post-NREM, spikes in ±1-s peri-SWR window were collected. The firing rate histograms (1-ms time bin) were constructed. The firing rate histogram for each unit was *z*-scored (based on the firing rate during 0.5 to 1 s before the SWR onset) and smoothed using a Gaussian kernel (SD = 20 ms), generating a PSTH. Only sessions with at least 50 SWRs (separated from other SWRs by at least 300 ms) were analyzed.

We followed a previously described procedure [55] to detect significantly SWR-modulated cells in RSC and CA1. Briefly, for each cell, we shuffled each spike train by a random amount up to ±0.5 s to generate 1,000 shuffled PSTHs. We then calculated the summed squared difference of the real PSTH relative to the mean of the shuffles in a ±200-ms window for RSC cells (in a 0- to 100-ms window for CA1 cells) around the SWR onset and compared it to the same score for each shuffle relative to the mean of the shuffles. The significance level of *P* < 0.05 indicated that the actual modulation exceeded 95% of the shuffles.

The calculation of ripple size in the SWR was similar to that reported in a previous publication [31]. High-amplitude ripples had a peak amplitude exceeding 10 SD relative to the mean, while mid- and low-amplitude ripples had peak amplitudes exceeding 6 and 4 SD below the mean, respectively.

### Ripple feature analysis

To determine which characteristics of ripple were influenced by chemogenetics, the ripple duration, number, and peak normalized power were calculated using buzcode-master and compared between CNO and vehicle group by Wilcoxon rank-sum test.

### GLMs for sleep periods

We constructed GLMs with log link functions to predict neural activity from ensemble activities in RSC or CA1 within specific time windows [10]. Only post-sleep sessions with at least 50 SWRs (separated from other SWRs by at least 300 ms) were analyzed. We calculated the spike count in a particular bin (100 ms) relative to the onset of the SWRs, yielding a vector of spike counts across cells (at least 5 cells) as predictor data. The predictor data in different time bins were used to predict single-cell SWR response (spike counts). Only cells that spiked in more than 50 SWRs were used as predictors or predicted cells. We performed a 5-fold cross-validation GLM approach when SWRs were randomly divided into 5 datasets with equal sizes. For each fold, we quantified the performance by calculating the average absolute difference between the predicted and accurate spike counts, defined as the prediction error. The mean baseline prediction errors were calculated after randomly shuffling spike counts 100 times in the same test fold. Prediction gain for each predicted cell in one window was calculated by the ratio of the mean baseline data error and the mean real data error across the 5 folds. As a control, we carried out 100 random shuffles of the original set and repeated the same process described above, corresponding to employing the predictor data for one SWR to predict the spike count for another SWR. To evaluate prediction significance, we compared the distribution of real prediction gains and the distribution of shuffled prediction gains within a given time window using a 2-tailed Wilcoxon rank-sum test.

To predict the occurrences of SWRs, we used a cross-validated binomial GLM approach. We divided all RSC spike trains into 100-ms bins for each post-sleep epoch and created a vector of 0/1 with the same length (1 indicating that the SWR occurred in the bin). We also applied 5-fold cross-validation with the constraint of the number of SWRs. Similarly, we calculated the real and shuffled prediction gains to quantify the performance of predicting the cell count. Shuffled prediction gains were computed by shuffling the occurrences of SWRs (the shuffled count of SWRs was consistent with the actual data). We compared the distribution of real prediction gains and the distribution of shuffled prediction gains within a given time window using a 2-tailed Wilcoxon signed rank test.

### OFF period detection

Only the sleep sessions with at least 5 RSC neurons were selected to detect the OFF period. Then, we identified the OFF period with no RSC spike firing for more than 100 ms. OFF periods with intervals <5 ms were merged.

### Putative cell type classification

Hierarchical clustering, as described in a reported study [31], was used to classify the retrosplenial isolated units as wide (putative excitatory) and narrow (putative inhibitory) spiking based on features of their average waveforms in the post-sleep session after single-unit tracking (see below). The average waveforms were normalized by the peak-to-trough amplitude. PCA was conducted based on normalized spike waveforms −0.2 ms to 0.8 ms relative to the trough. After extracting the 3 major principal components (PCs), we used a hierarchical clustering algorithm to find the similarity (Euclidean distance) between all pairs of normalized waveforms in PC space, then generated a hierarchical binary cluster tree, and identified 3 clusters from the hierarchical tree. While classifying CA1 cells, we followed the processing pipeline in CellExplorer-master to classify them into 2 putative cell types: narrow interneurons (putative inhibitory neurons, trough to peak ≤ 0.425 ms) and pyramidal cells (putative excitatory neurons trough to peak > 0.425 ms and acg_tau_rise ≤ 6 ms, Ref cellexplorer paper).

### Single-unit tracking

To identify the same unit in pre-/post-sleep and task sessions, we adopted the previous approach [22] by matching single units between 2 discontinuous recordings. We matched the pre-/post-sleep sessions and task/post-sleep sessions, respectively, and then found the intersection to track single units in 3 sessions. Taking matching units in pre-/post-sleep sessions as an example, each isolated unit in the pre-sleep data (defined as “Pre”) had candidate matches in the post-sleep recording (defined as “Post”) from the same channels. A similarity score was calculated for all “Pre” units on these candidate “Post” units based on 2 categories of similarity metrics derived from the average spike waveform features:

1. Local spike features

Three parameters were calculated to compare the specific features of the average “Pre” and “Post” waveforms, including the similarities in the trough-to-peak duration, the trough-to-peak amplitude, and the peak-to-trough amplitude (the amplitude difference between the trough and the first peak before the trough). The similarities in these features were calculated by the difference function:

$$\text{Similarit}y\left(\mathrm{pre},\text{post}\right)=\max\left(0,1-\left|\frac{\mathrm{pre}-\text{post}}{\mathrm{pre}}\right|\right)$$

2. Global spike features

To quantify the temporal similarities between the entire average spike waveform (1-ms window, from −0.2 ms to 0.8 ms relative to the trough) of the “Pre” and the candidate “Post” units, the following 3 parameters were used after aligning the waveforms with dynamic time warping (DTW) between all pairs of average waveforms or normalized average waveforms: (a) spike waveform similarity, defined as 1 minus max difference (max difference is the maximum Euclidean distance between the dynamic time-warped average waveforms normalized by the peak-to-trough amplitude); (b) spike waveform correlation, defined as the Pearson correlation coefficient of “Pre” versus “Post” dynamic time-warped average waveforms; and (c) DTW scale factor, defined as the ratio of the length of the original “Pre” average spike waveform to the length of the signal after DTW, to penalize excessive waveform warping.

We calculated the L2 norm of the above categories’ similarity metrics. The weights were empirically assigned as W_local_ = 1/5 and W_global_ = 4/5, similar to the previous method [22]. The final similarity score was defined as the sum of 2 weighted metrics. For each “Pre” unit, the “Post” unit was tagged as the best candidate match if all the criteria were met: (a) the highest similarity score, (b) spike waveform correlation ≥ 0.95, (c) similarity (trough-to-peak duration) ≥ 0.7. If a given “Post” unit had more than one potential matching “Pre” unit, it was only assigned to the one with the largest similarity score. Lastly, all the matching pairs were manually checked to delete the explicitly false matches according to the average waveforms. Only cells that were tracked during all 3 epochs were analyzed.

### Identifying location-related cells and splitters

Spikes were recruited when the mouse’s running speed was higher than 3 cm/s to exclude puzzling behaviors such as eating and grooming. The maze was divided into 0.36 cm × 0.36 cm bins to characterize firing fields and firing rate distributions. Cells with >200 spikes per session were included for location-related analyses. Spike counts and time maps were smoothed with a 2-dimensional Gaussian kernel over the neighboring 5 × 5 bins. Spatial firing rate maps were calculated by dividing the smoothed map of spike numbers by time. We identified location-related cells with at least one robust place field, an area (7 × 7 adjacent bins) with at least 40 bins, each having a firing rate at least twice the mean rate (dividing the number of spikes by the time in the entire session). Previous works have found that the RSC neurons can encode various information, including motor, spatial, and cue information [22,24,25]. To avoid the influence of motion and other confusing factors, we analyzed the firing patterns of RSC cells in the stem. To study RSC location-related cells that can distinguish the routes to the left and right reward locations, especially before the turns, we divided the stem of the maze into 3 subareas (10 cm long × 6 cm wide per subarea) and calculated the firing rate within each subarea for each trial. Incorrect trials were excluded from all analyses. The firing rate within each subarea was defined as the number of spikes divided by the amount of time spent in the subarea. To canvass differential firing on the stem in the correct left trials and correct right trials for each cell, a 2-way ANOVA was run with subarea (3 levels) and trial type (go-left and go-right trials) as independent variables and the firing rate as the dependent measure. We identified cells whose firing rates showed a significant main effect of trial type or interaction of trial type and 3 subareas as splitters (potentially distinguishing go-left and go-right trials). Moreover, the location-related cell only with a significant main effect of subarea but no significant main effect of trial type nor a trial type × 3 subareas interaction was identified as a conventional location-related cell. Meanwhile, to avoid the effect of speed, we identified speed cells as follows and discarded the putative speed cells (only 9 cells). The center position of the animal in each frame was represented in Cartesian coordinates, and the instantaneous locomotion speed was calculated as the ratio between the distances traveled in 2 contiguous frames. Consistent with the previous study on RSC speed cells [22], we regarded the average speed firing rate for each cell as a speed function and calculated as the spike counts divided by the time spent in each speed bin (1 cm/s bin). Periods when the animal moved at speeds >5 cm/s and <35 cm/s were analyzed due to low sample sizes at high speeds. The speed score was defined as the absolute Pearson correlation (*P* < 0.01) between the average speed firing rate (a function of speed) and the animal’s locomotion speed. Cells were classified as speed-tuned if their speed scores were higher than the 99th percentile of the shuffled distribution, generated for each cell by following the shuffling procedure with 100 permutations. For each bout of the shuffling process, the spike trains from each cell were created by shifting a random period between 10 and 30 s along the animal’s trajectory and the session length minus the random offset, with the end wrapped to the beginning of the session. This shuffling procedure retained the temporal firing patterns in the original data but disturbed the spatial characteristics.

### Histology

At the end of the experiments, mice were anesthetized with isoflurane gas and transcardially perfused with phosphate-buffered saline (PBS), followed by 4% paraformaldehyde (PFA) solution. Brains were removed and stored successively in 4% PFA for at least 24 h and 30% sucrose for at least 36 h and then frozen and sectioned in a cryostat (25-μm coronal sections, Leica). The brain slices were Iba1 stained (1:800, Abcam, ab48004), Nissl stained (1:1,000, Invitrogen N21480), and coverslipped. Lastly, we employed the research slide scanner (VS200, OLYMPUS) to show the putative recorded or lesion location.

For immunofluorescence staining of CTB retrograde tracing, mice were transcardially perfused and brains were processed as described above. Brain sections were first washed in PBS and then blocked in PBST (0.5% Triton X-100 in PBS) containing 5% bovine serum albumin for 1.5 h at room temperature and incubated overnight at 4 °C in the same blocking solution containing appropriate primary antibody (mouse anti-CaMKII, 1:600, Abcam, catalog no. ab22609; rabbit anti-GAD65/67, 1:500, Sigma, catalog no. G5163). The next day, brain sections were washed with PBST for 3 times (10 min each) and incubated with appropriate secondary antibodies (donkey anti-mouse Alexa Fluor 647, Invitrogen, catalog no. A31571, 1:800; donkey anti-rabbit Alexa Fluor 488, Invitrogen, catalog no. A32790, 1:800) at room temperature for 1.5 h. Brain sections were counterstained with 4′,6-diamidino-2-phenylindole (DAPI) (10 μg/ml, Solarbio, catalog no. C0065). After washes in PBST for 3 times (10 min each), brain sections were coverslipped with mounting media (Fluoromount-G, SouthernBiotech, catalog no. 0100-01).

## Data Availability

All data are available from the corresponding author upon request.
