# Supplementary material for: Coordinated Interactions between the Hippocampus and Retrosplenial Cortex in Spatial Memory
Source: Research (Wash D C). 2024 Oct 31;7:0521. doi: 10.34133/research.0521 (PMC11525046; doi:10.34133/research.0521)
Supplement: Supplementary 1 — Figs. S1 to S11 [file research.0521.f1.zip › Figure S4.pdf]

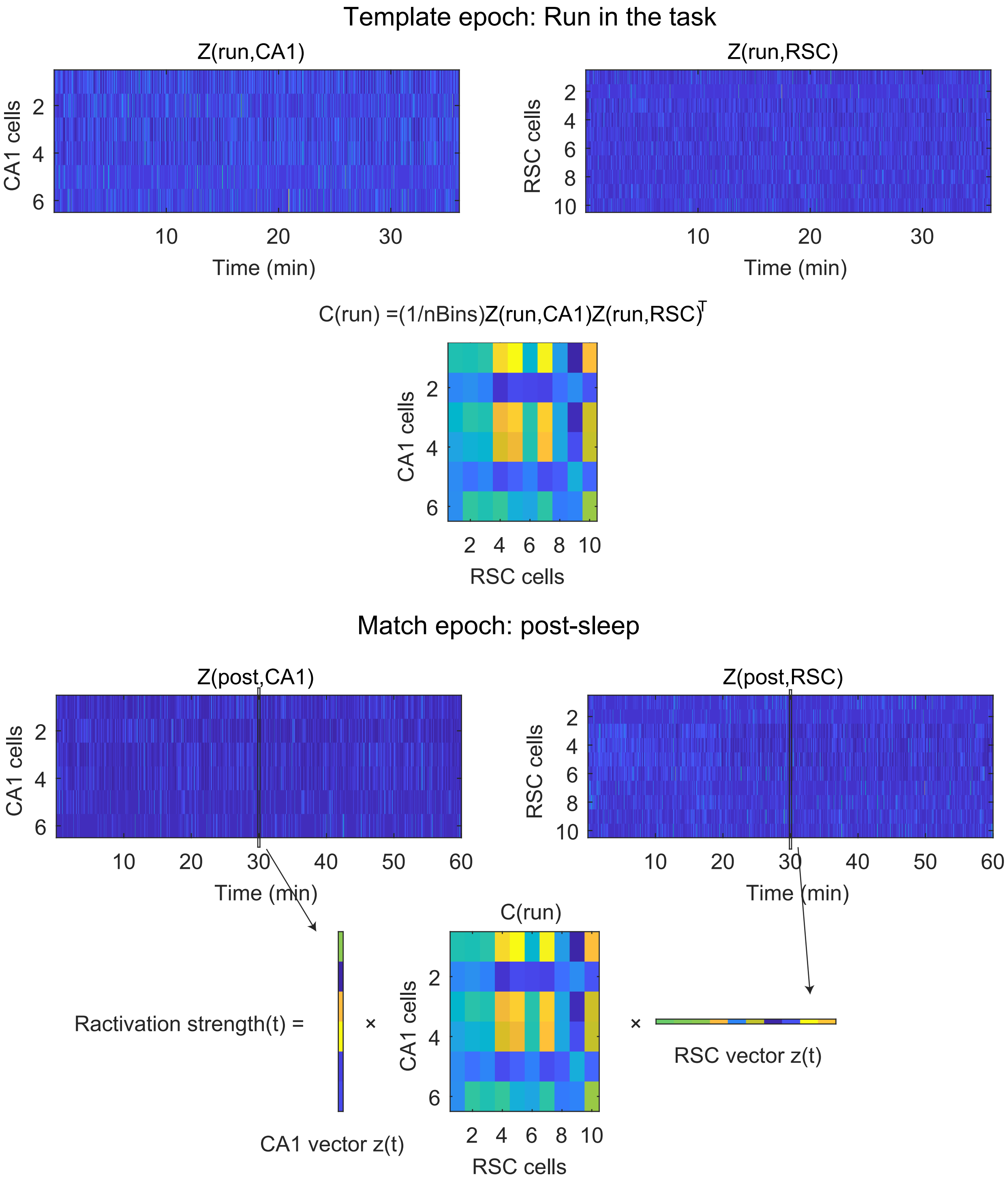

Template epoch: Run in the task
Z(run,CA1)
Z(run,RSC)
CA1 cells
RSC cells
Time (min)
C(run) =(1/nBins)Z(run,CA1)Z(run,RSC)T
Match epoch: post-sleep
Z(post,CA1)
Z(post,RSC)
C(run)
Ractivation strength(t) =
×
×
RSC vector z(t)
CA1 vector z(t)
