# Supplementary material for: Coordinated Interactions between the Hippocampus and Retrosplenial Cortex in Spatial Memory
Source: Research (Wash D C). 2024 Oct 31;7:0521. doi: 10.34133/research.0521 (PMC11525046; doi:10.34133/research.0521)
Supplement: Supplementary 1 — Figs. S1 to S11 [file research.0521.f1.zip › Supplementary Materials.pdf]

Supplementary Materials

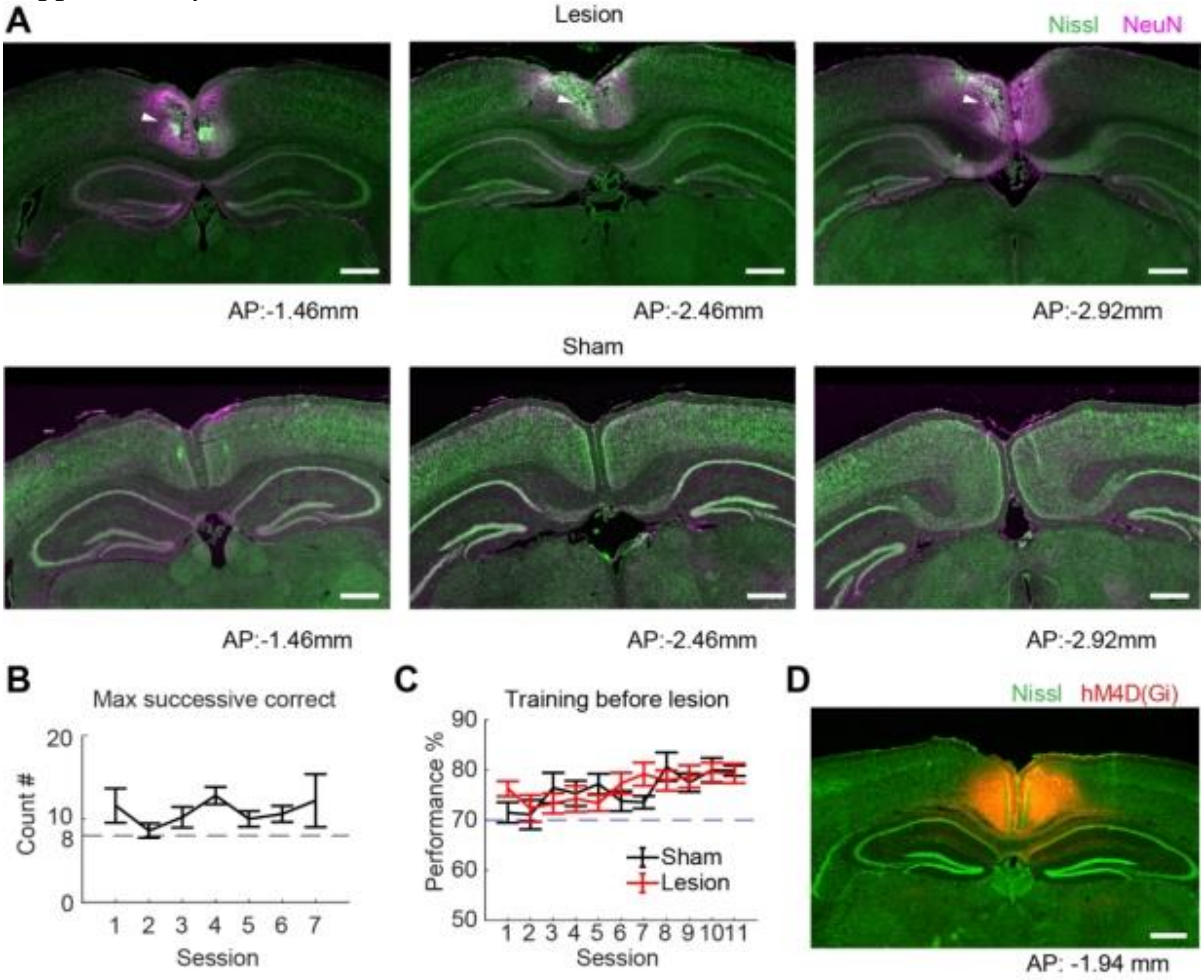

Fig. S1 Recording or lesion sites and behavioral performance before the lesion.
(A) Top: coronal brain sections showing the damaged areas (white arrow) with NMDA lesions of the RSC at 3 AP positions. Bottom: coronal brain sections with sham operation of the RSC at 3 AP positions. Scale bars: 500 µm.
(B) Maximum successive correct during the training phase (n = 5), with a criterion line at 8.
(C) Behavioral performance during training phase before lesion (lesion: n = 8, sham: n = 7, $F_{(10,143)} = 0.91$, $p = 0.52$, two-way ANOVA).
(D) Coronal brain section showing the expression of chemogenetic virus (hSyn-hM4D(Gi)-mCherry). Green: Nissl staining; Red: hSyn-hM4D(Gi)-mCherry. Scale bars: 500 µm.

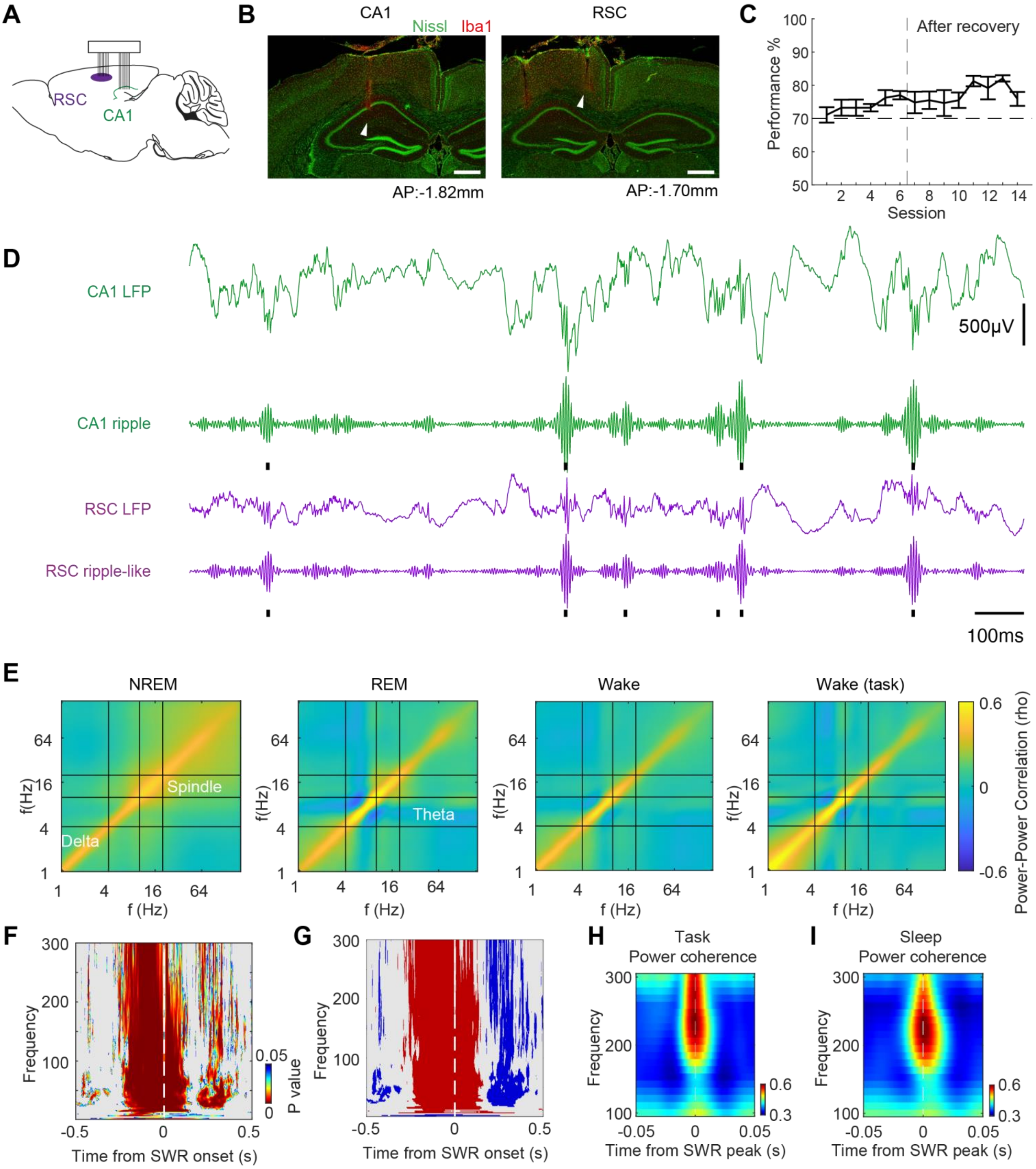

Fig. S2. CA1-RSC correlation in different brain states.
(A) Schematic drawing of dual-site multi-tetrode recording in the hippocampus and RSC.
(B) Coronal brain sections showing the recording site (white arrow) in the hippocampal CA1 (left) and RSC (right). Scale bars: 500 μm.
(C) Behavioral performance across sessions before and after surgery (n=5).
(D) Representative LFPs and ripple like raw data. The short black vertical lines indicate the time points of CA1 ripples or RSC ripple-like events.
(E) Comodulograms (power-power spearman correlation) of LFP during waking state (in sleep/task phase), NREM, or REM sleep demonstrate power synchronization in some bands.

(F) The difference in the power of frequency band around SWR onset between the task and sleep phases in RSC. Areas where differences are significant ($p<0.05$) are highlighted in color, while non-significant ($p>0.05$) areas shown in gray, based on one-way ANOVA.
(G) The difference in the power of frequency band around SWR onset between the task and sleep phases in RSC, Area with significant higher ($p<0.05$) power is in red, while area with significant lower power ($p<0.05$) is in blue, based on one-way ANOVA.
(H) Coherogram of wavelet power between CA1 and RSC LFPs centered on CA1 SWR peaks during the task.
(I) Corresponding analyses to G for CA1 and RSC neural data during the sleep phase.

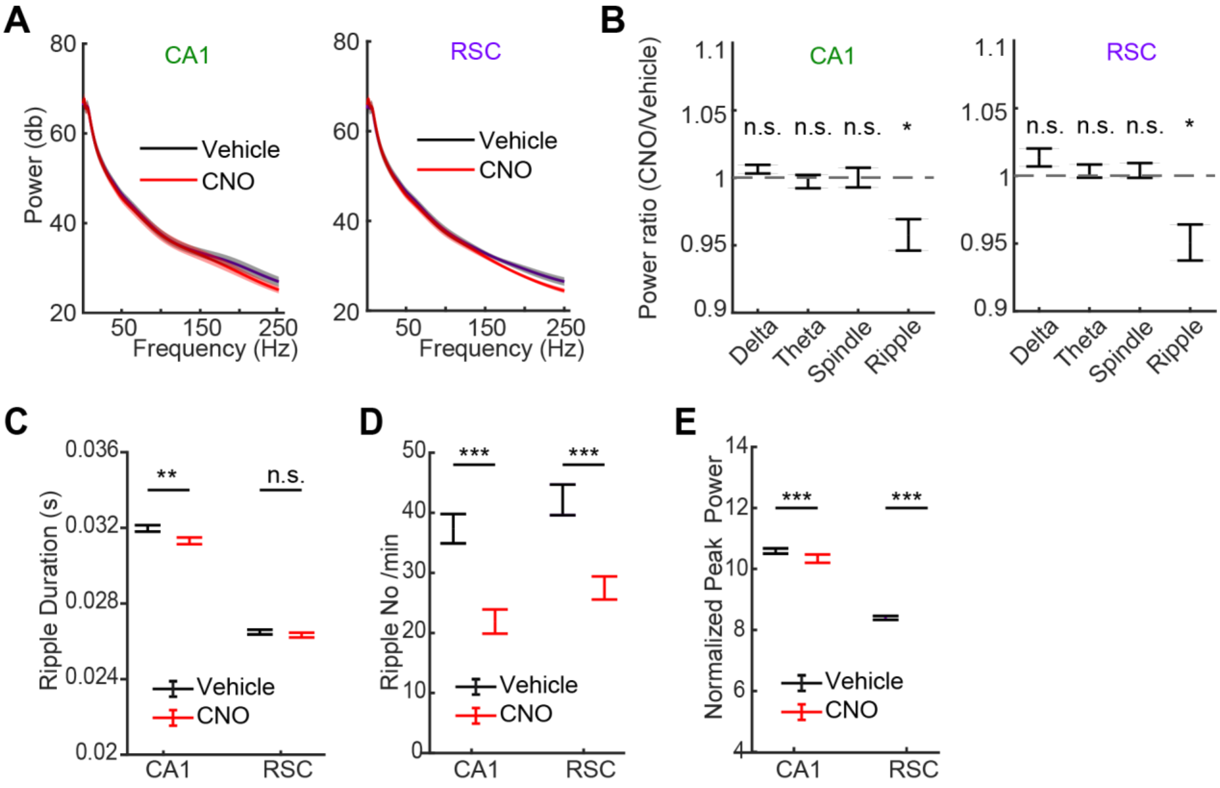

Fig. S3 Ripple oscillation between hippocampus and RSC during SWRs with chemogenetic manipulation.
(A) Average power spectral densities during NREM sleep in CA1 (left) and RSC (right) following chemogenetic manipulation.
(B) Comparative analysis of CNO versus vehicle in power spectral densities across specific frequency bands: delta (1-4Hz), theta (6-10Hz), spindle (10-20Hz), and ripple (140-250Hz) within CA1 (left) and RSC (right). *$p<0.05$, Wilcoxon signed rank test.
(C to E) Analysis of ripple characteristics in CA1 (left) and RSC (right): Ripple duration (G), number of ripples per minute (H), and normalized peak power (I). **$p<0.01$, ***$p<0.001$, based on Wilcoxon rank-sum test.

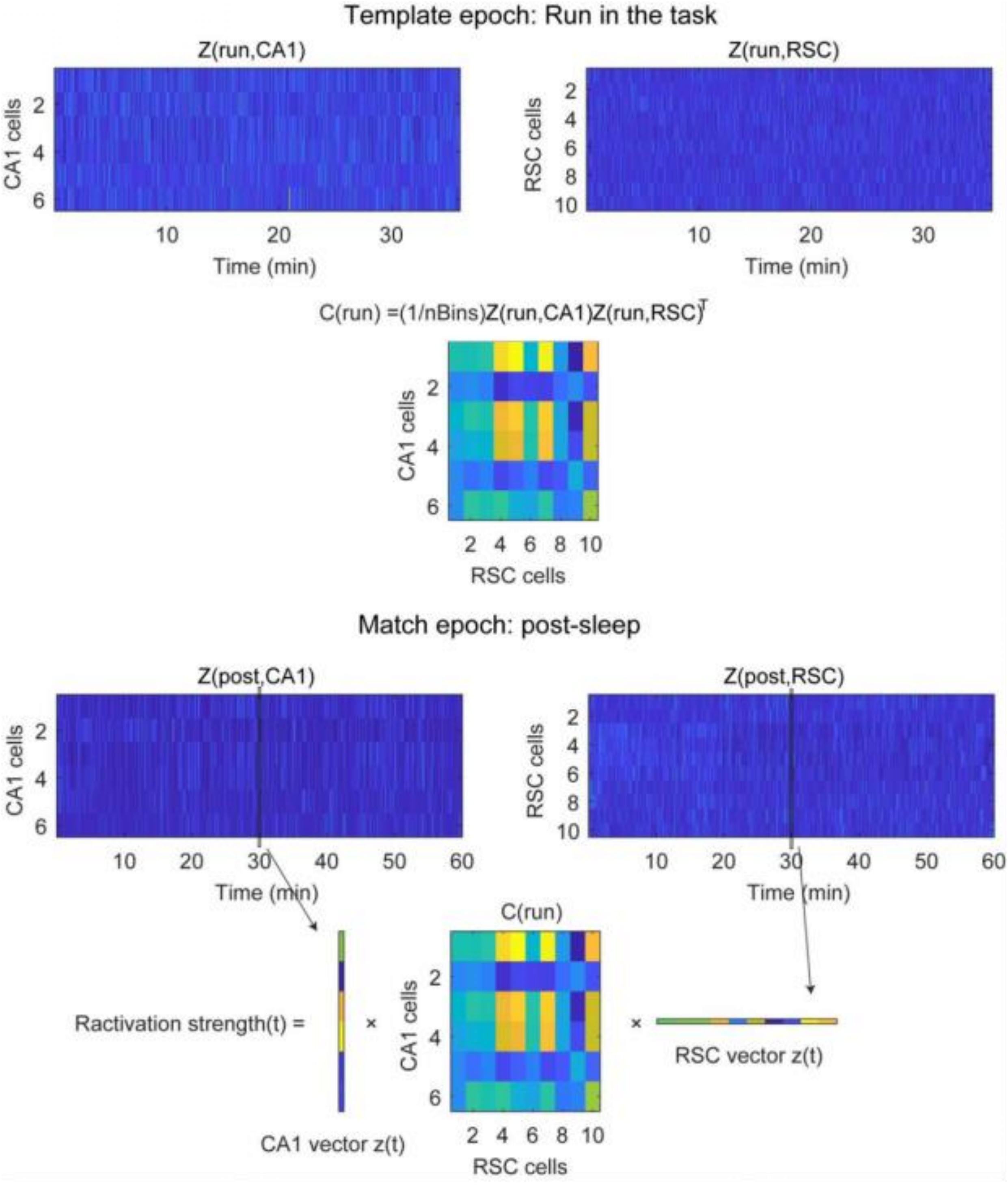

Fig. S4 Reactivation strength: method summary.
A template correlation matrix is computed for all CA1-RSC pairs based on the binned and z-scored spike trains during the run within the spatial task. The reactivation during each time bin of the match phase (post-sleep) is calculated as the match between the global correlations during running (C(run)) and the CA1-RSC correlation in a time bin t.

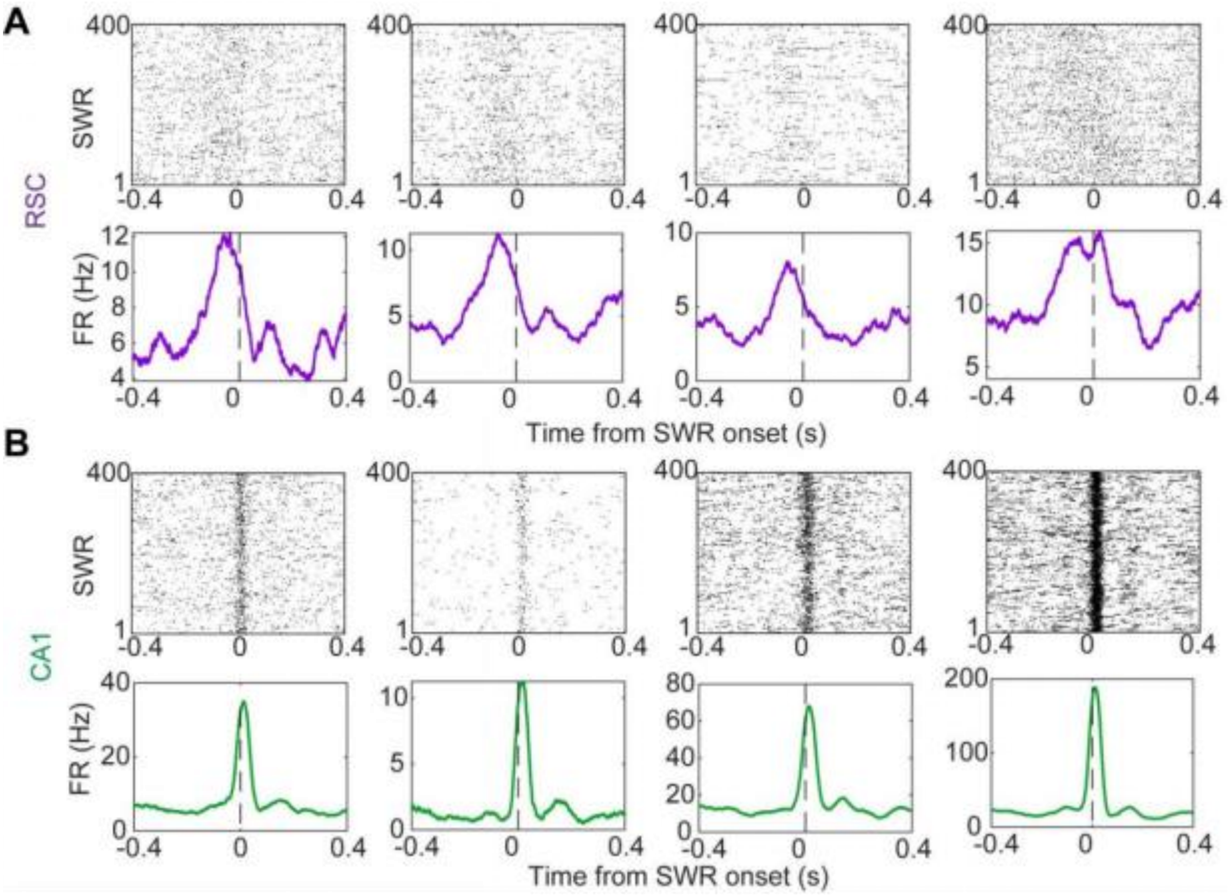

Fig. S5 Examples of SWR-related activity during sleep.
(A) SWR-aligned rasters of four significantly SWR-modulated RSC example neurons and corresponding time histograms. FR, firing rate.
(B) SWR-aligned rasters of four significantly SWR-modulated CA1 example neurons and corresponding time histograms. FR, firing rate.

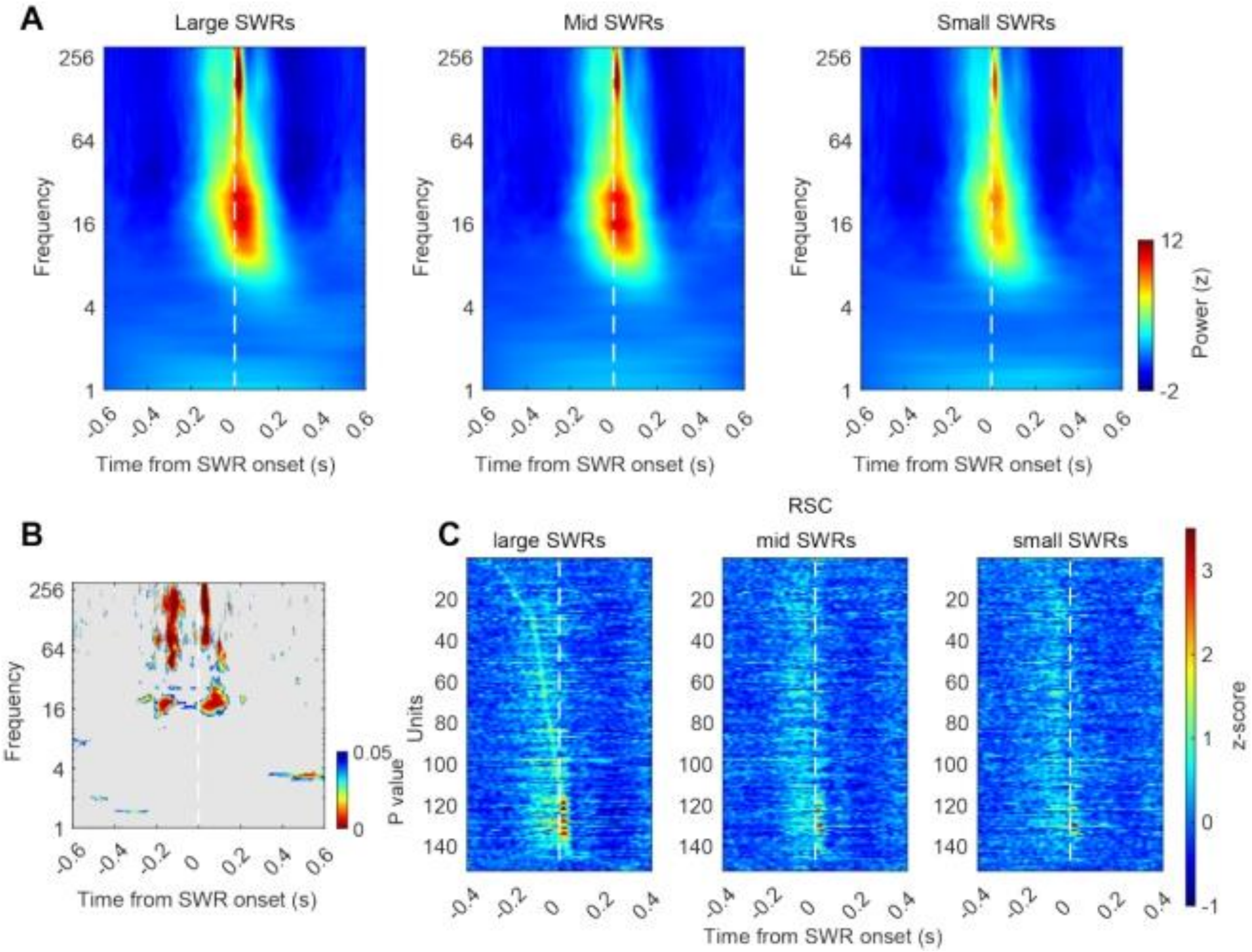

Fig. S6 RSC activity during sleep was correlated with hippocampal SWR size.
(A) Mean spectrograms from RSC locked to the onset of CA1 SWRs in relation to different sizes of SWR events (left, large ripples; intermediate, middle ripples; right, small ripples) during post-NREM.
(B) The difference in the power of each frequency band at each lag around SWR onsets among different sizes of SWR events (large, middle, and small amplitude ripples) in RSC. One-way ANOVA, $p<0.05$. Non-significant ($p>0.05$) areas are shown in gray.
(C) Z-scored SWR-related PSTHs of all modulated RSC neurons in relation to different sizes of SWR events (left, large ripples; intermediate, middle ripples; right, small ripples) in post-NREM sleep.

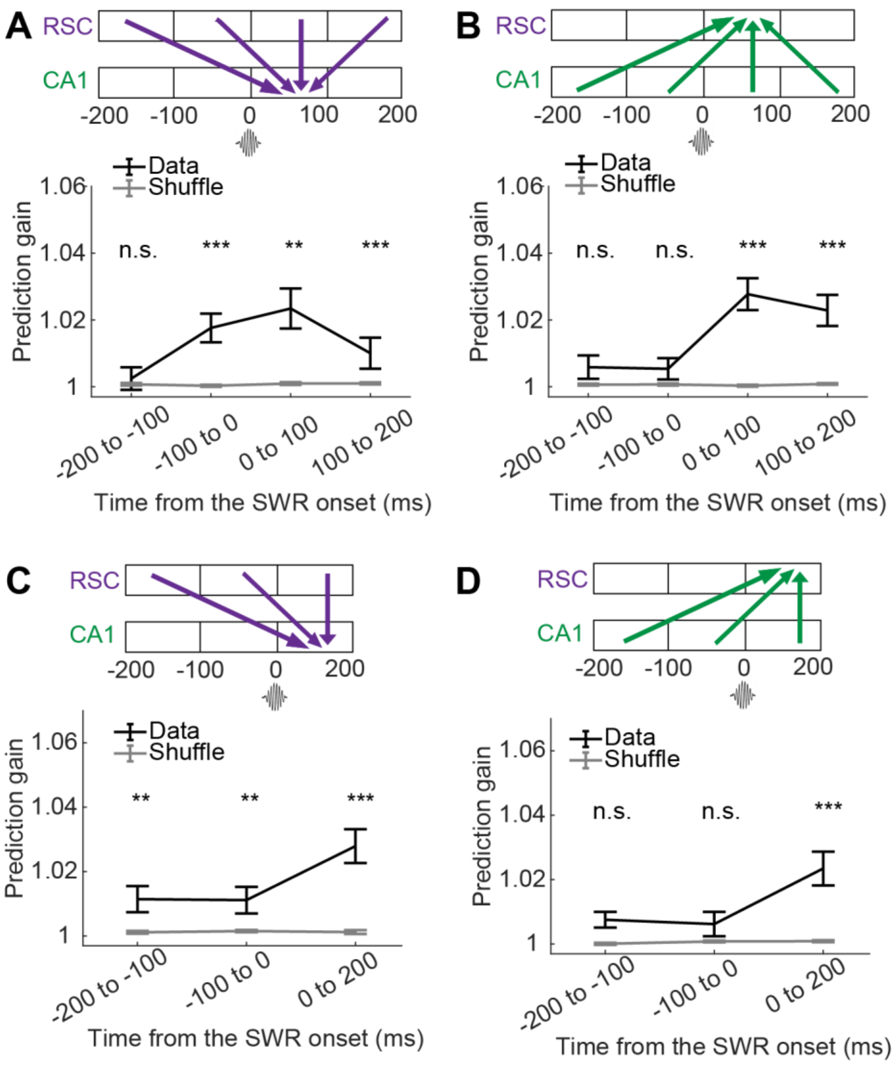

Fig. S7 Hippocampus–RSC interaction around SWRs during sleep.

(A) Prediction of CA1 neuron spiking during SWRs based on RSC ensemble patterns over various time windows (n = 76 predicted CA1 cells). **$p<0.01$, ***$p<0.001$ vs shuffle.

(B) Prediction of RSC neuron spiking during SWRs based on CA1 ensemble patterns (n = 100 predicted RSC cells). ***$p<0.001$ vs shuffle.

(C) Similar to A, Predicting CA1 Single-Cell Spiking During 0-200ms. **$p<0.01$, ***$p<0.001$ vs shuffle.

(D) Similar to B, Predicting RSC Single-Cell Spiking During 0-200ms. ***$p<0.001$ vs shuffle.

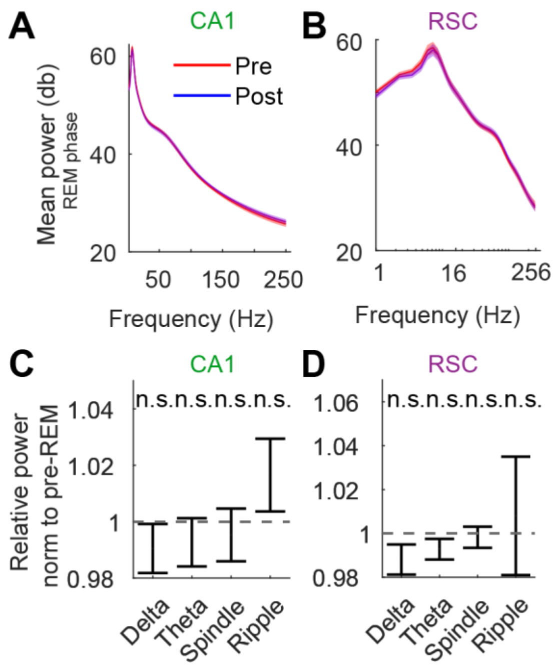

Fig. S8 No significant changes in oscillation power were observed in RSC and CA1 during REM sleep.
(A-B) average power spectral densities during REM phase in CA1 (A) and RSC (B).
(C-D) ratios of pre-REM to post-REM average power spectral density in delta(1-4Hz), theta(6-10Hz), spindle(10-20Hz), and ripple(140-250Hz) bands in CA1 (C) and RSC (D).

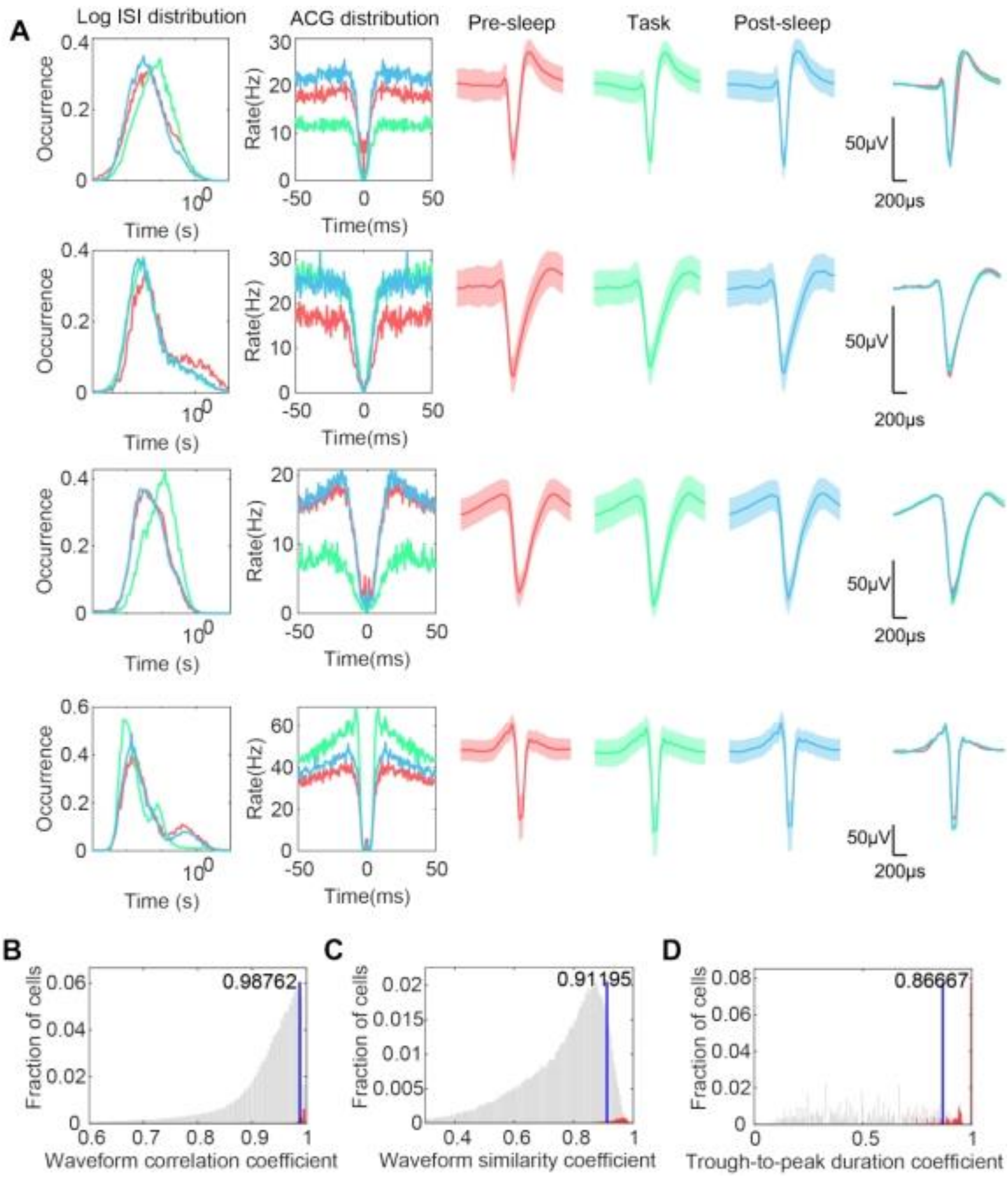

Fig. S9 Physiological characterizations of CA1 and RSC neurons across three recording phases

(A) Log inter spike interval (ISI) distribution and autocorrelogram (ACG) distribution of examples of tracing the same neuron in three recording phases (pre-/post-sleep and spatial task). Red, green, and blue correspond to pre-sleep, task, and post-sleep phases, respectively.

(B) Distribution of waveform correlation for all pairs. The blue stippled lines mark the 90th percentile significance level of the distribution. The red bars indicate the distribution for the matched pairs.

(C) Distribution of spike waveform similarity for all pairs.

(D) Distribution of the similarity of trough-to-peak duration for all pairs.

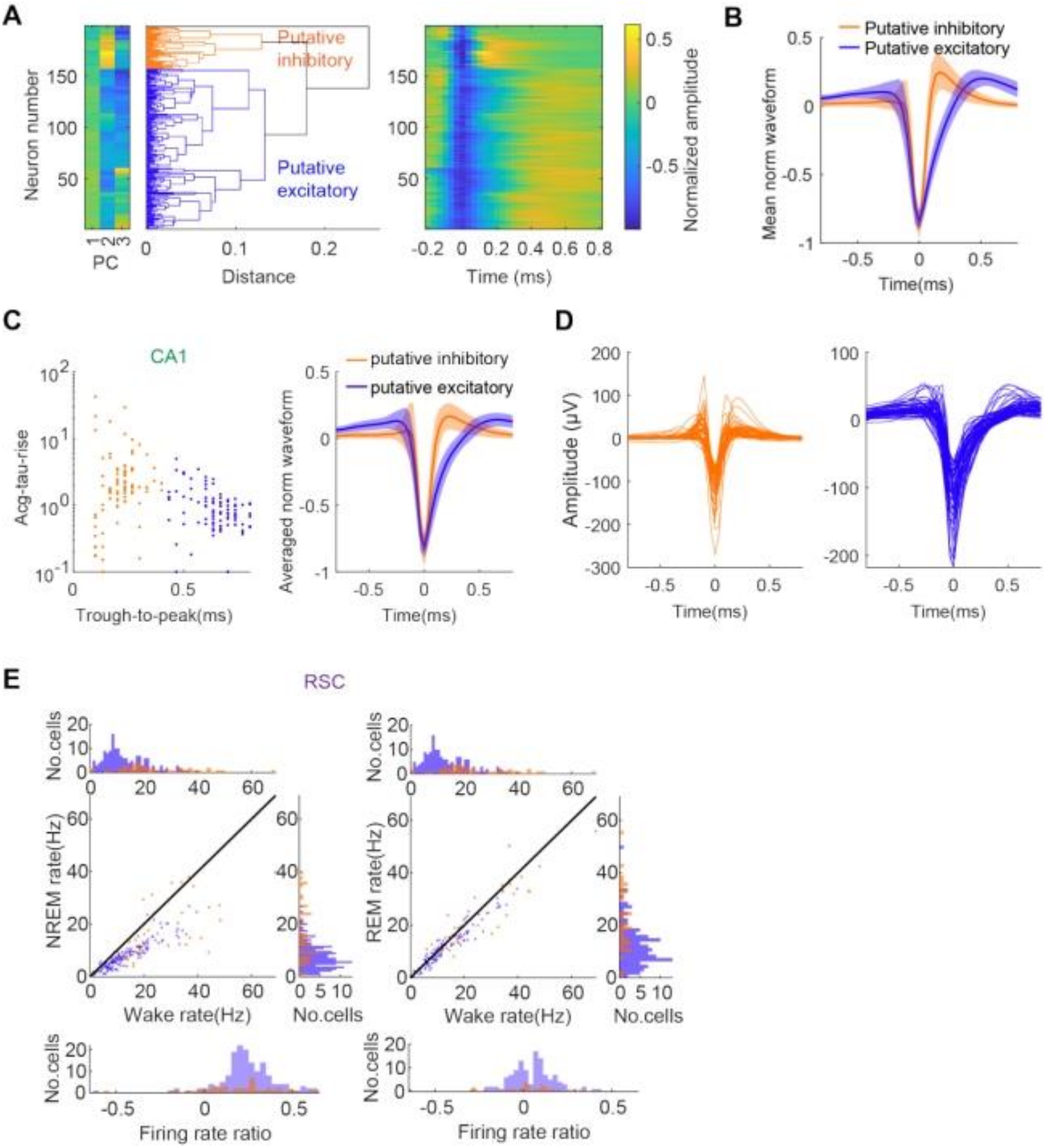

Fig. S10 Physiological characterizations of putative inhibitory and excitatory neurons in CA1 and RSC across three recording phases.

(A) RSC Neuron Classification and Waveform Analysis. Principal-component analysis (PCA) segregating RSC neurons into putative inhibitory (orange; n = 42) and excitatory (blue; n = 158) categories. Principal components PC1, PC2, and PC3 are color-graded from low (blue) to high (yellow) scores. Right: Heatmap displaying spike waveforms for all RSC neurons. The order of neurons corresponds to the arrangement in the left panel.

(B) Averaged normalized spike waveforms of RSC neurons. Orange and blue correspond to putative inhibitory and excitatory neurons, respectively.

(C) Classification and Waveform Analysis of CA1 Neurons. Left: Classification of recorded CA1 neurons into identified inhibitory (orange, n = 93) and excitatory (blue, n = 97) based on trough-to-peak time and autocorrelogram (ACG) tau-rise metrics. Right: Averaged normalized spike waveforms for CA1 neurons.

(D) Original waveform of CA1 inhibitory (Left) and excitatory (Right) neurons.

(E) Firing Rate Distributions and Ratios in RSC. Top: Comparative distributions of firing rates for identified inhibitory and excitatory neurons in RSC during non-rapid eye movement (NREM) sleep versus wakefulness (top left) and rapid eye movement (REM) sleep versus wakefulness (top right). Bottom: Distributions of firing rate ratios (NREM/wake and REM/wake) for inhibitory (left) and excitatory (right) neurons in RSC.

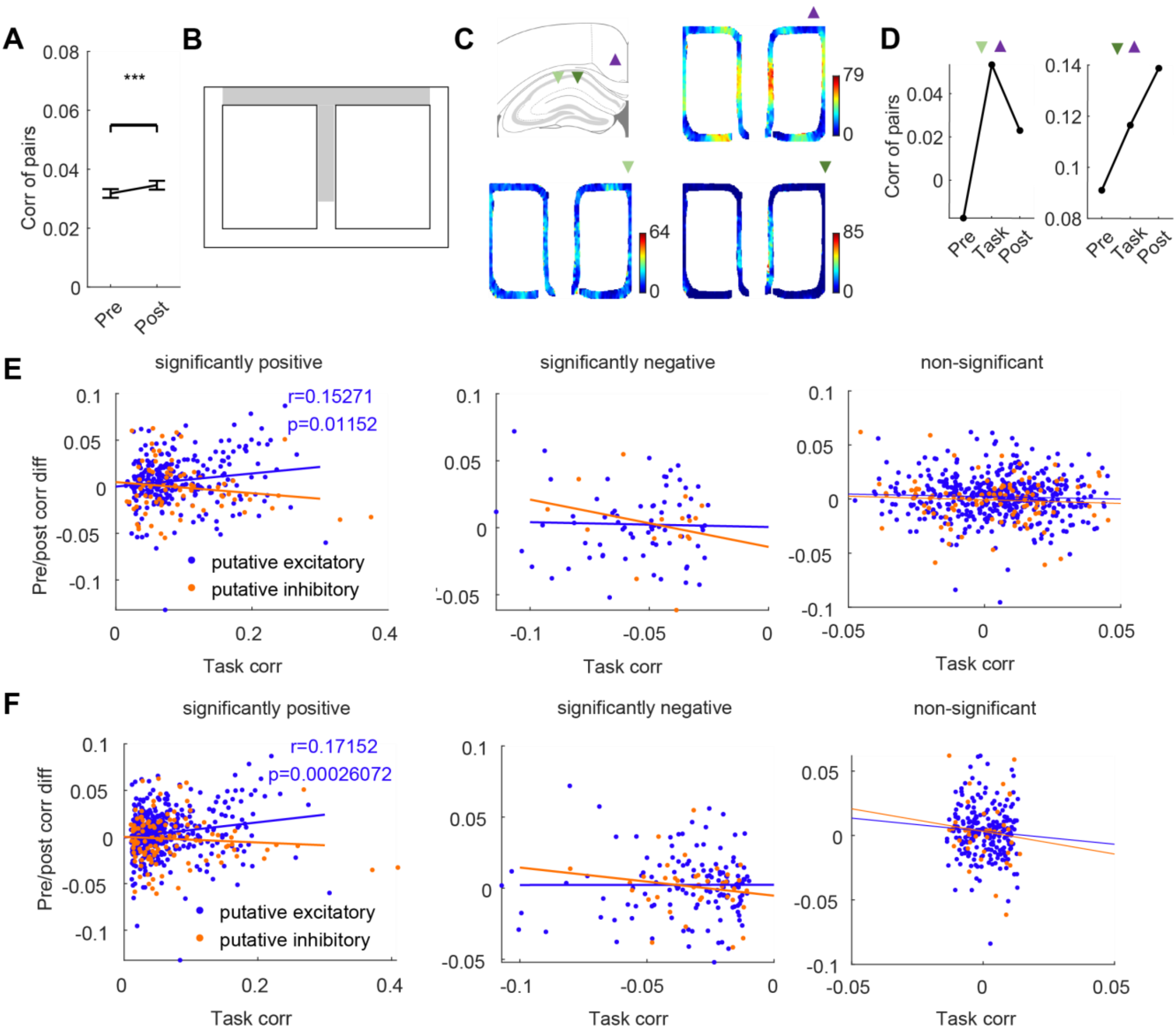

Fig. S11 The correlations of CA1-RSC cell pairs in task and NREM sleep

(A) The temporal correlations of all CA1-RSC pairs are shown for pre-NREM and post-NREM phases. ***p<0.001, sign-rank test.

(B) Schematic of the figure-eight maze indicating the approaching trajectory in the stem and side arms (gray shaded area, defined as Task phase) used in the correlation of CA1-RSC pairs analyses in spatial task phases.

(C) Behavioral correlates of an example RSC putative excitatory neuron (top right, violet triangle) that forms significantly positive Task correlation pairs with two CA1 neurons (bottom, pale green, and dark green triangles). Firing heatmaps are shown for the run phase. Color bars are in hertz.

(D) Pearson's correlation analysis for example neuron pairs for the two example pairs from (C) across pre-sleep, Task, and post-sleep phases.

(E) Pearson’s correlation of CA1-RSC pairs correlations for approaching area shown in B. Left, significantly positive correlation pairs in Task phases; middle, extremely negative correlation pairs in Task phases; right, non-significant correlation pairs. Orange and blue correspond to putative inhibitory and excitatory neurons, respectively.

(F) Similar to E, but for the entire spatial task area.
