# Supplementary figures and images for: Coordinated Interactions between the Hippocampus and Retrosplenial Cortex in Spatial Memory
Source: Research (Wash D C). 2024 Oct 31;7:0521. doi: 10.34133/research.0521 (PMC11525046; doi:10.34133/research.0521)

**A**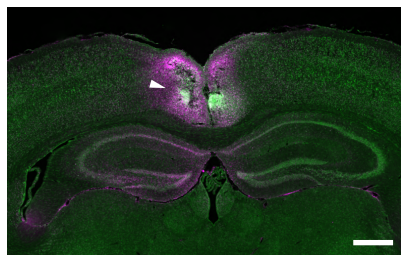

AP: -1.46mm

Lesion

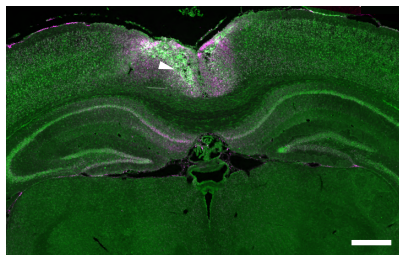

AP: -2.46mm

Nissl NeuN

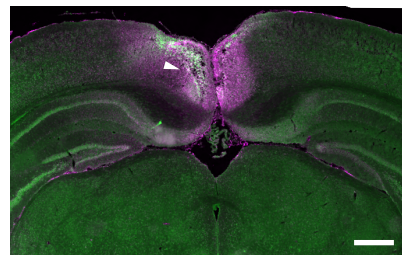

AP: -2.92mm

Sham

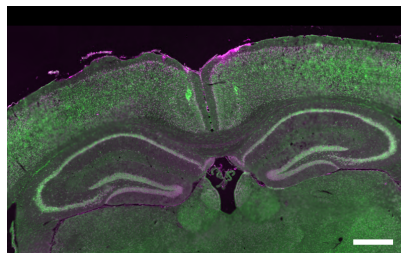

AP: -1.46mm

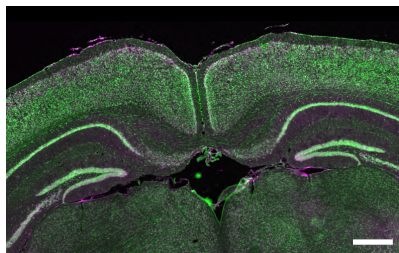

AP: -2.46mm

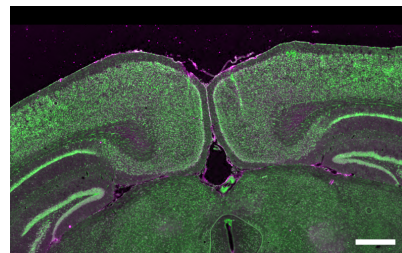

AP: -2.92mm

**B**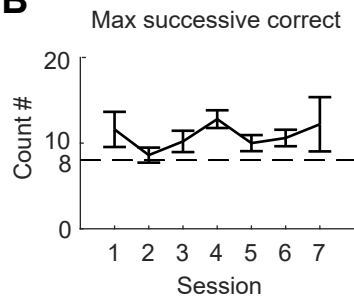**C**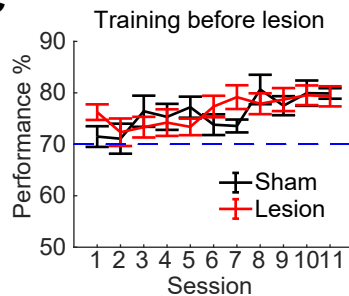**D**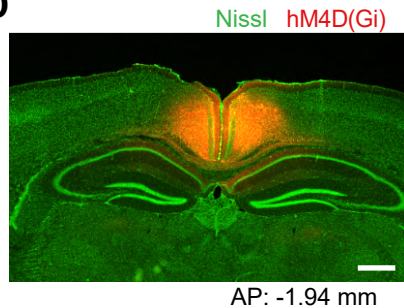

AP: -1.94 mm

Supplement: Supplementary 1 — Figs. S1 to S11 [file research.0521.f1.zip › Figure S1.pdf]

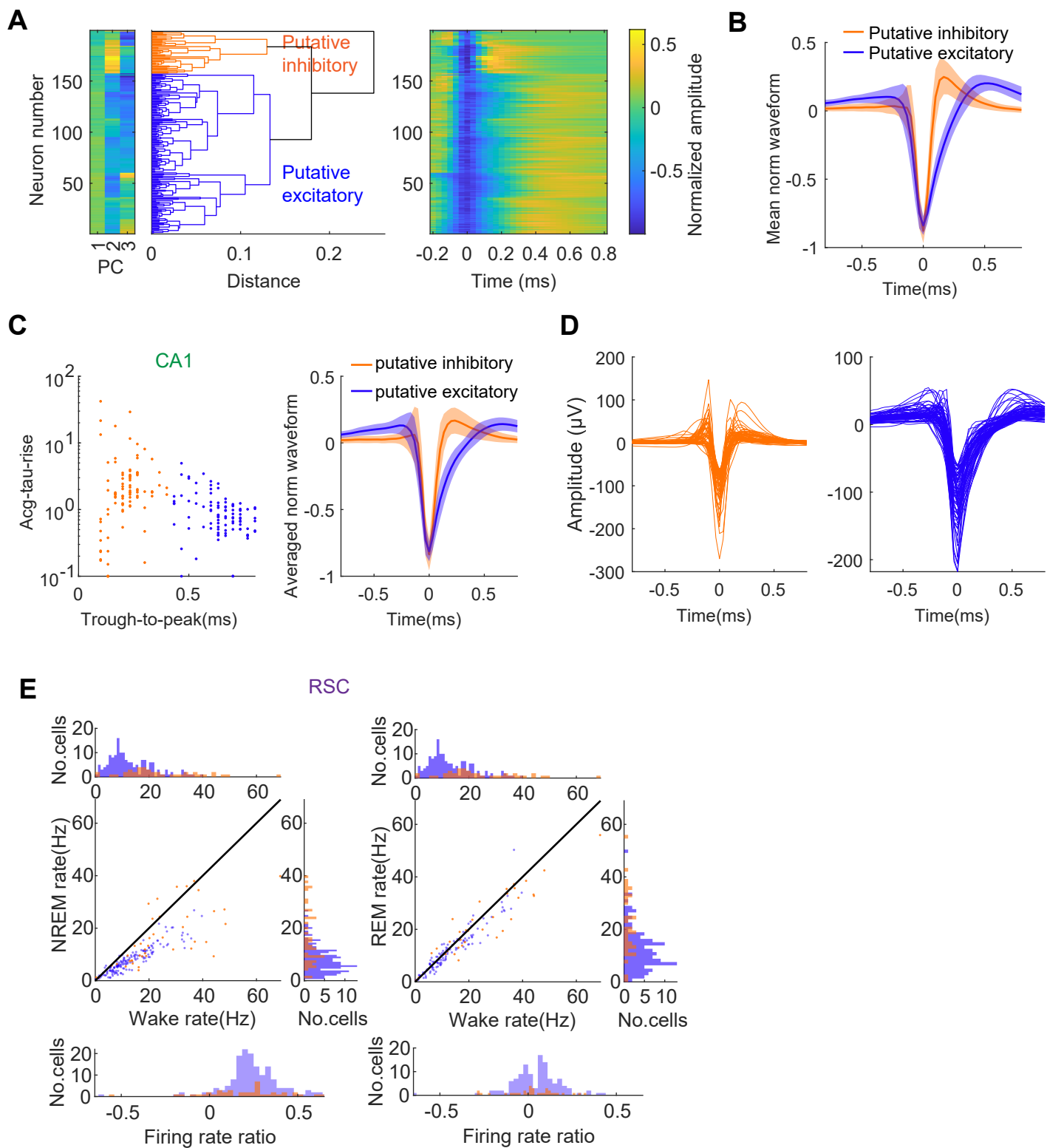

Supplement: Supplementary 1 — Figs. S1 to S11 [file research.0521.f1.zip › Figure S10.pdf]

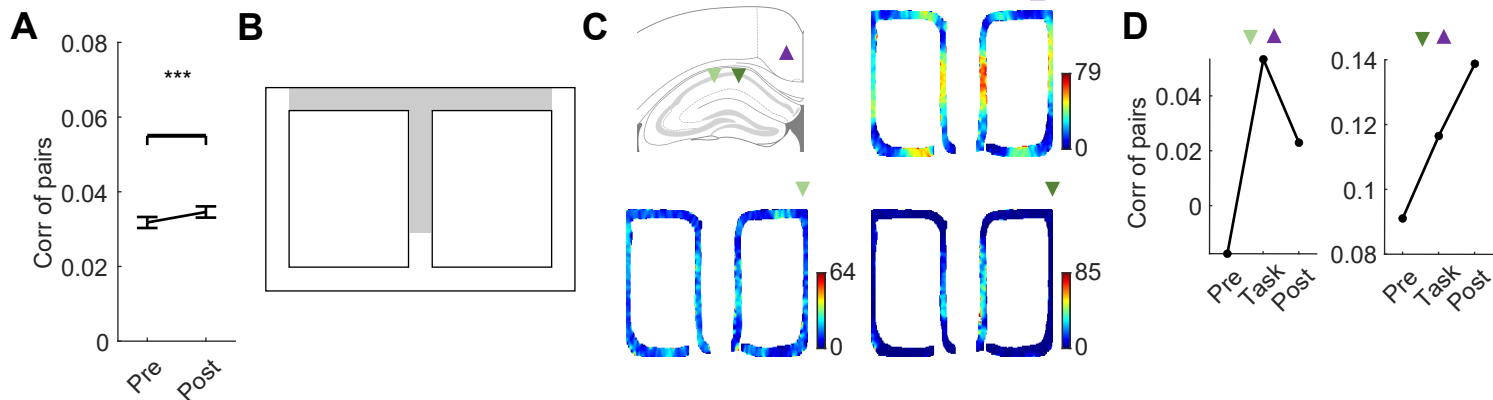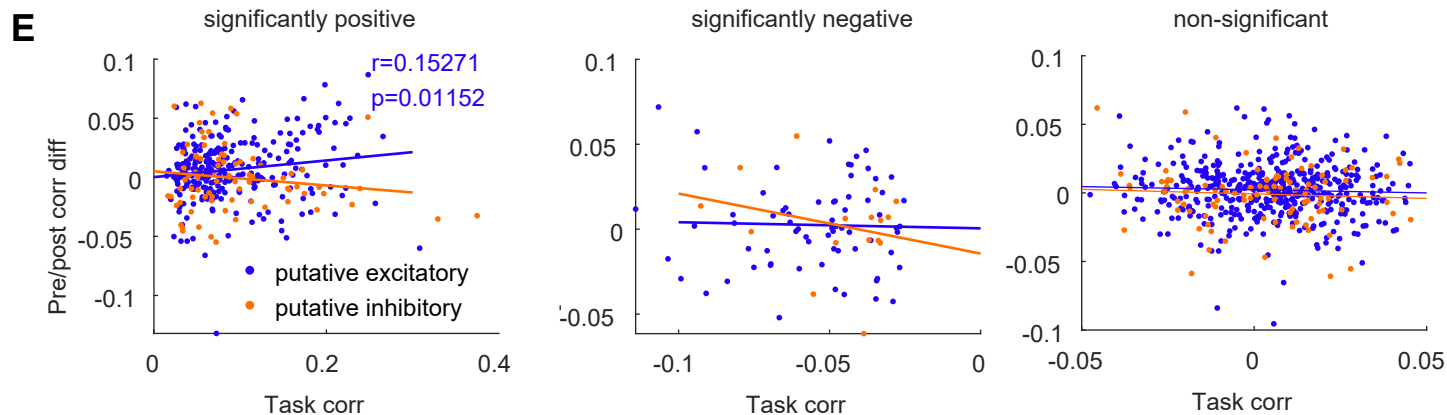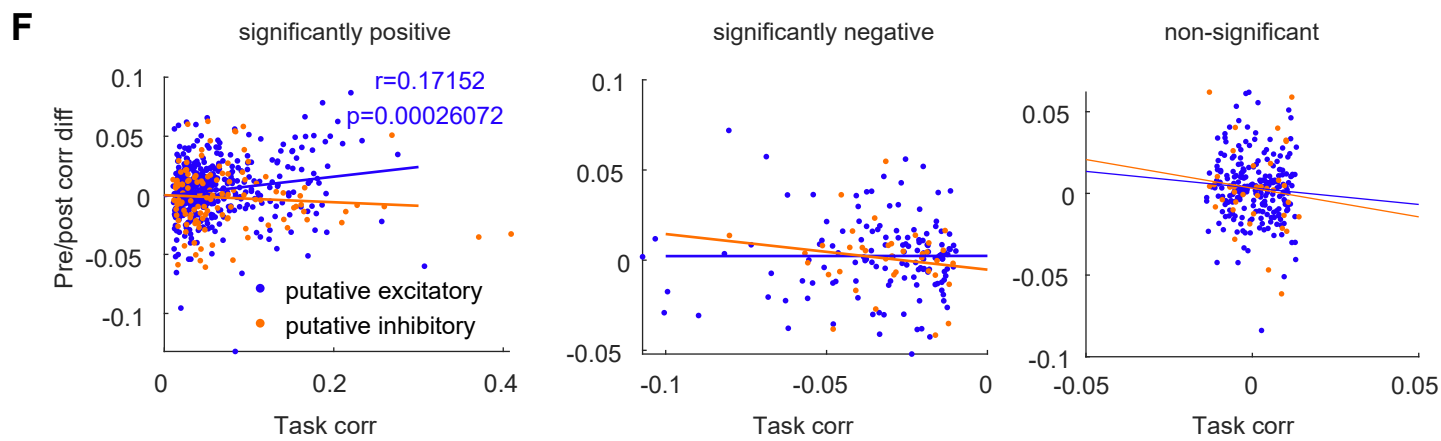

Supplement: Supplementary 1 — Figs. S1 to S11 [file research.0521.f1.zip › Figure S11.pdf]

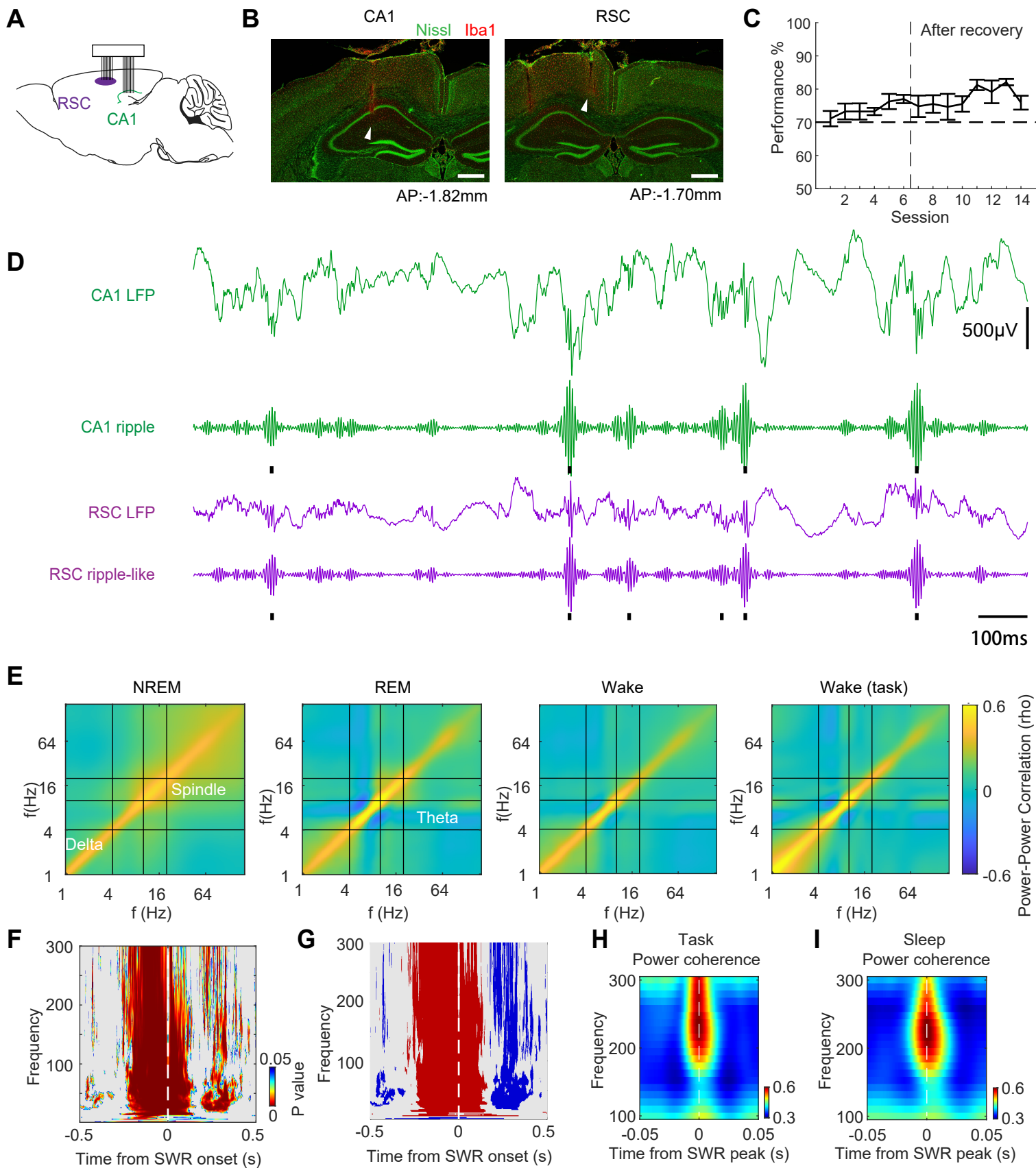

Supplement: Supplementary 1 — Figs. S1 to S11 [file research.0521.f1.zip › Figure S2.pdf]

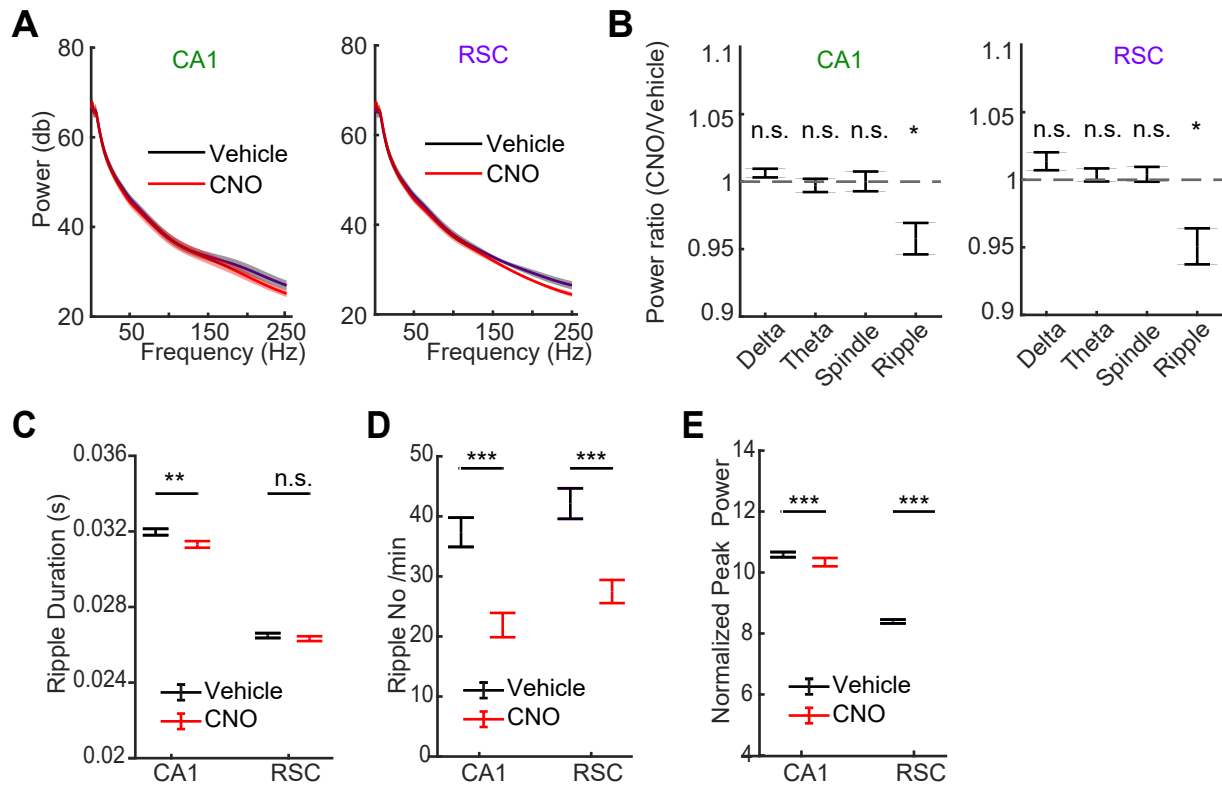

Supplement: Supplementary 1 — Figs. S1 to S11 [file research.0521.f1.zip › Figure S3.pdf]

**A**

RSC

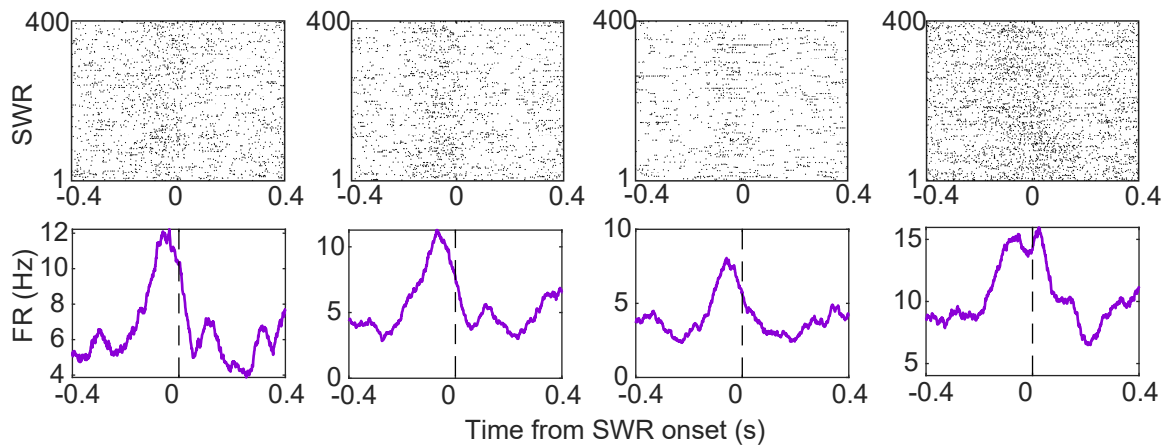**B**

CA1

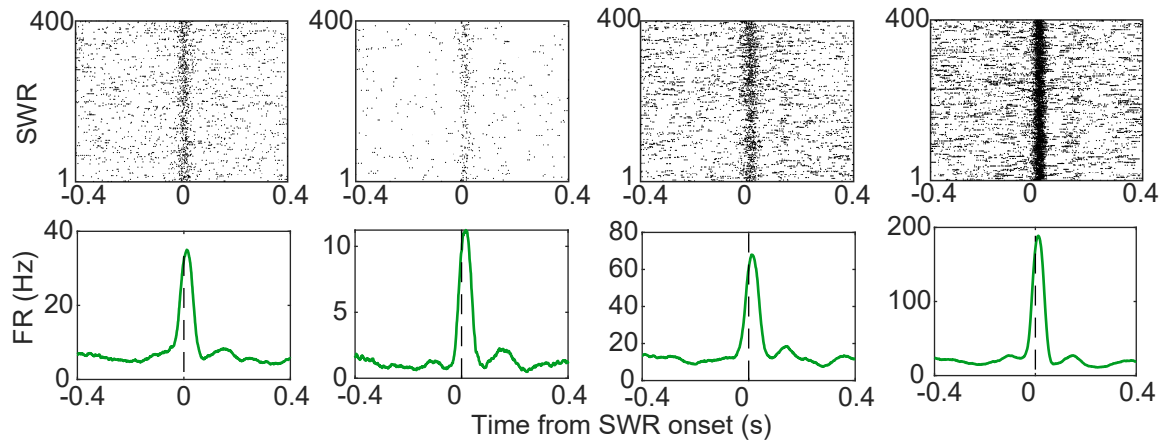

Supplement: Supplementary 1 — Figs. S1 to S11 [file research.0521.f1.zip › Figure S5.pdf]

**A**

Large SWRs

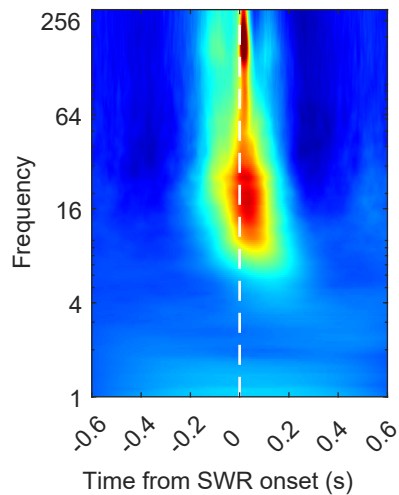

Mid SWRs

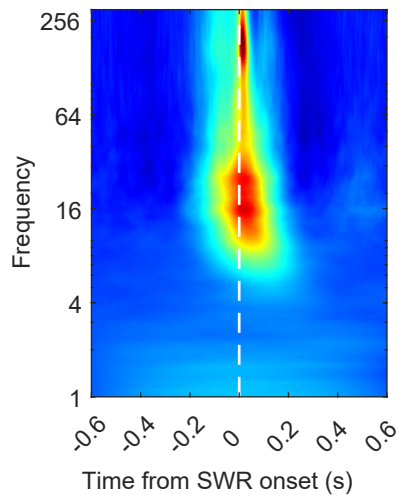

Small SWRs

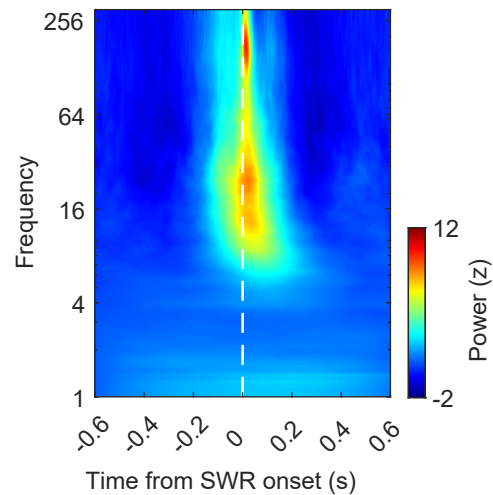**B**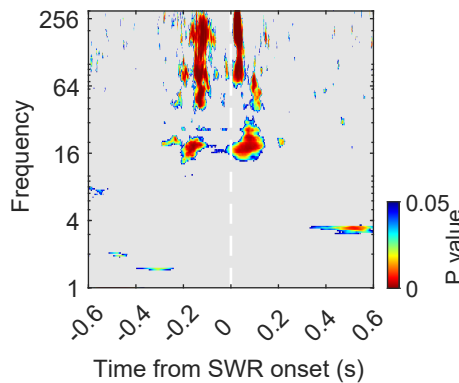**C**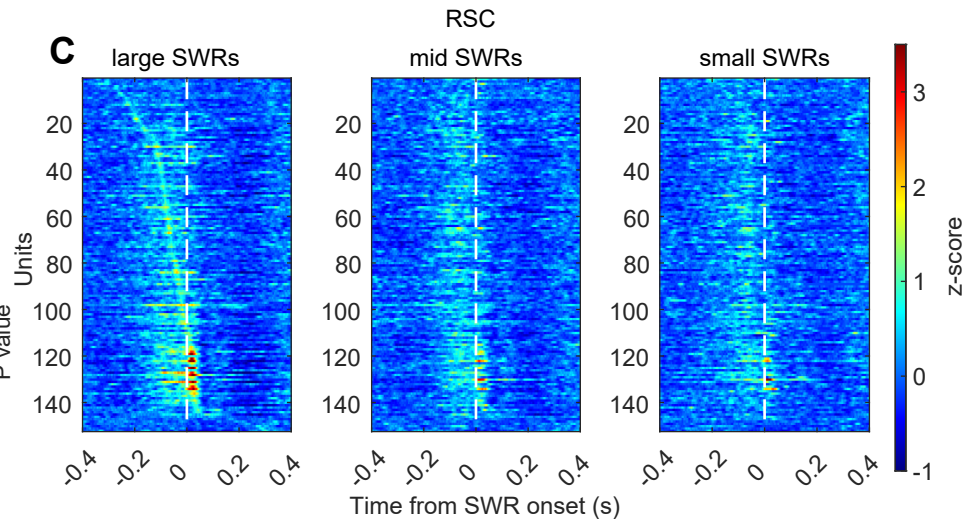

Supplement: Supplementary 1 — Figs. S1 to S11 [file research.0521.f1.zip › Figure S6.pdf]

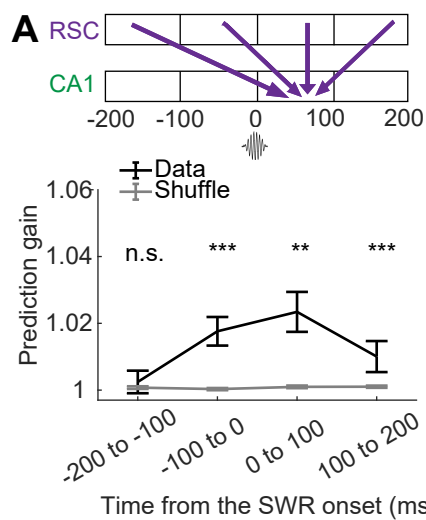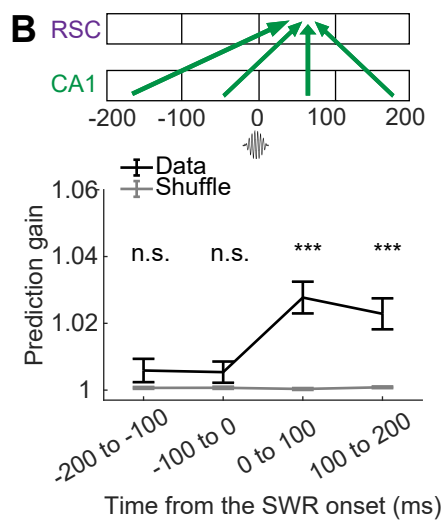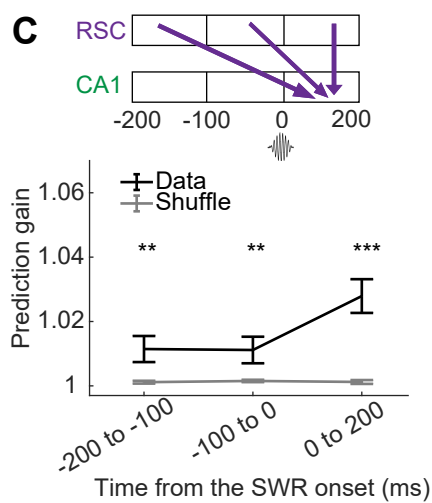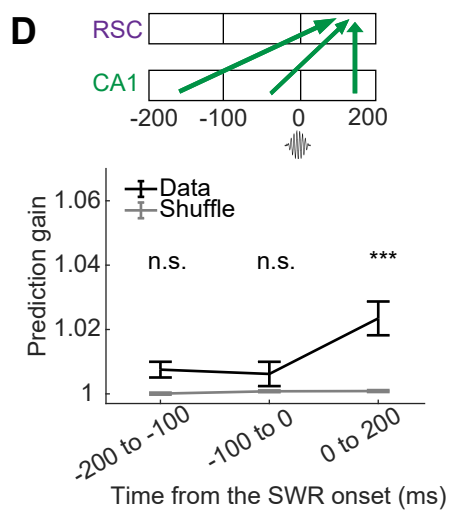

Supplement: Supplementary 1 — Figs. S1 to S11 [file research.0521.f1.zip › Figure S7.pdf]

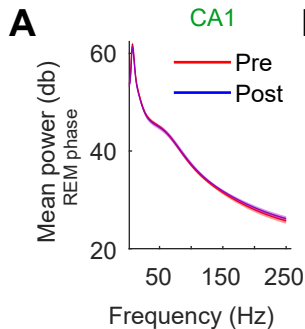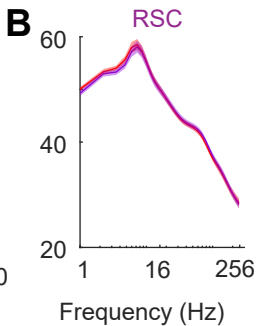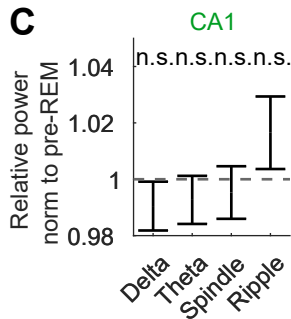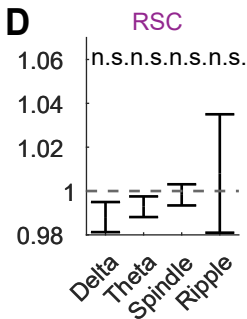

Supplement: Supplementary 1 — Figs. S1 to S11 [file research.0521.f1.zip › Figure S8.pdf]

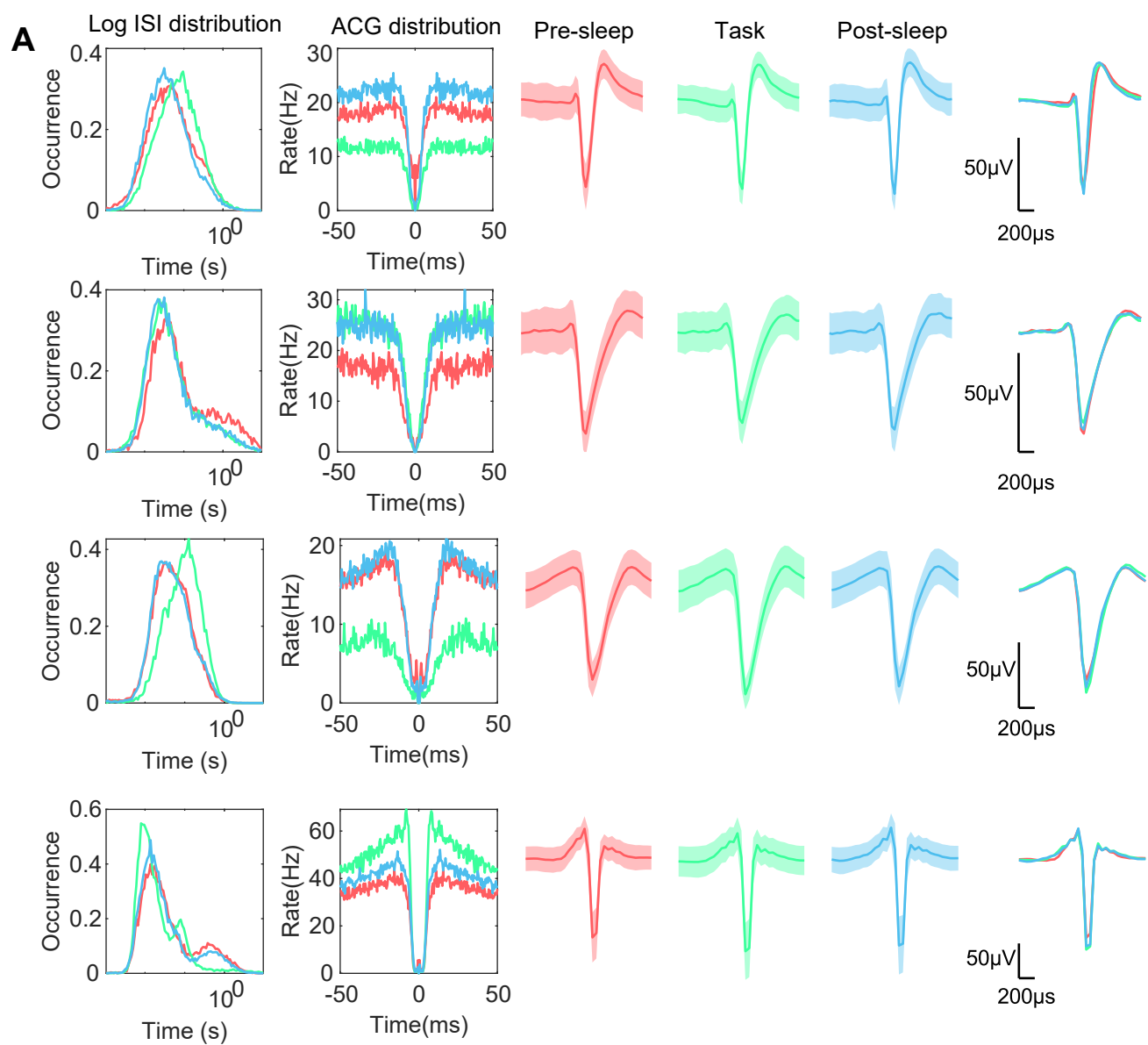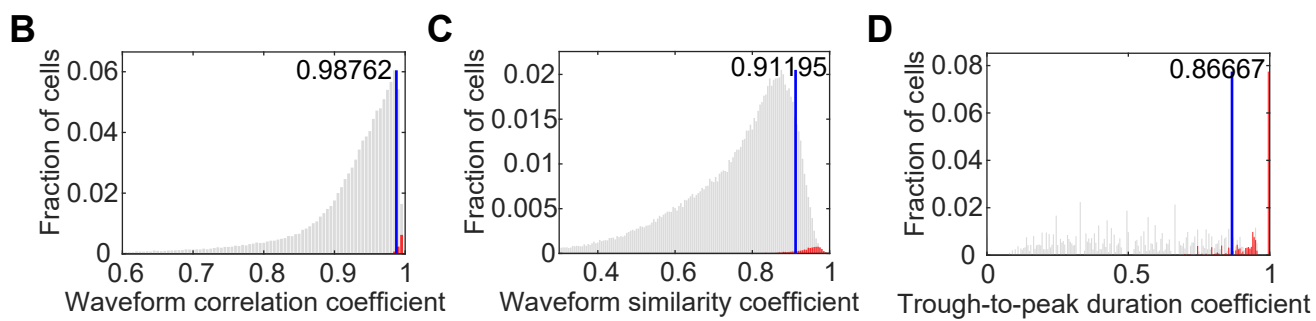

Supplement: Supplementary 1 — Figs. S1 to S11 [file research.0521.f1.zip › Figure S9.pdf]
